# Synthesis, SAR, and in silico studies of new benzochromene derivatives as insecticidal agents against *Culex pipiens* L. larvae and adults

**DOI:** 10.1038/s41598-025-30027-z

**Published:** 2025-12-09

**Authors:** Hossam Ghonim, Wael S. I. Abou-Elmagd, Eid M. Khalil, Abdelaal A. Abdalha, Sandy S. Samir, Doaa R. Abdel-Haleem, Mohamed H. Hekal

**Affiliations:** 1 Department of Chemistry, Faculty of Science, Ain Shams University, Abbassia, Cairo, 11566 Egypt; 2 Chemistry Department, Faculty of Science, Helwan University, Ain Helwan, Cairo, 11795 Egypt; 3 Entomology Department, Faculty of Science, Ain Shams University, Abbassia, Cairo, 11566 Egypt

**Keywords:** Benzochromenes, Chromenotriazolo[1,5-c]pyrimidines, SAR study, In silico study, Larvicidal activity, Adulticidal activity, *Culex pipiens*, Biochemistry, Chemical biology, Drug discovery, Zoology

## Abstract

Mosquito control is a favorable strategy to prevent mosquito-borne diseases. There are various approaches for the management of potentially dreadful mosquito populations such as larvicides and adulticides. In this study, a series of novel benzochromene derivatives were synthesized and evaluated for their insecticidal efficacy against larvae and adult stages of *Culex pipiens* L. The synthesized compounds underwent comprehensive structural characterization and toxicological evaluation, revealing their larvicidal and adulticidal activity for several derivatives. The tested compounds were more effective against larvae than adults of *C. pipiens*. Compounds **10** and **5** exhibited pronounced larvicidal activity, surpassing the reference compound temephos, with LC₅₀ values of 40.15 and 46.17 ppm, respectively. In addition, both compounds displayed remarkable adulticidal efficacy, with LC₅₀ values of 55.02 and 65.43 ppm, respectively, whereas compound 6 showed the lowest potency among the tested series with LC₅₀ value of 215.40 ppm. Notably, compounds **10** and **5** were 5.36- and 4.66-fold more active against larvae than compound **6**, and demonstrated 5.28- and 4.44-fold higher adulticidal efficiency compared with deltamethrin. In cytotoxicity assays, compound **5** displayed an IC₅₀ value of 72.65 µM, while compound **10** showed an IC₅₀ of 90.32 µM, indicating low toxicity toward normal human cells and a favorable selectivity index. Molecular docking supported this observation by demonstrating strong and specific binding interactions with *Anopheles gambiae* acetylcholinesterase (AChE). Additionally, the DFT results provided insights into the electronic properties and stability of the compounds, while MEP mapping highlighted key reactive sites associated with target interactions. Structure–activity relationship (SAR) studies identified crucial functional groups influencing insecticidal potency.

**Supplementary Information:**

The online version contains supplementary material available at 10.1038/s41598-025-30027-z.

## Introduction

Several dreadful diseases transmitted by different mosquito species include dengue fever, Zika, chikungunya, yellow fever, Japanese encephalitis, filariasis and West Nile fever^1^. Mosquito females are obligate blood feeders on humans or animals, which is responsible for the transmission of pathogenic microbes to them^2^. Mosquito-borne diseases are an amplifying global health threat, affecting more than 40% of the world’s population and expected to spread the risk of arbovirus transmission to half of the global population by 2050^3^. *Culex pipiens* L. is a common species, widely distributed in tropical and subtropical areas. It is a vector of many diseases causing severe morbidity and consequently numerous mortalities to humans and animals, such as Rift Valley fever, West Nile fever and Bancroftian filariasis, which threaten over 100 million people annually^4,5^. The eradication of vector-borne diseases is primarily dependent on disrupting the transmission cycle of the disease. The most efficient approach for diminishing mosquito existence is targeting the aquatic larvae in their breeding sites or destroying aerial adults with the application of suitable pesticides^6,7^. The traditional approaches employed for mosquito control were the application of organophosphates and pyrethroids for adults and organophosphates to mosquito breeding sites for larvae^8^. Continual use of traditional insecticides has led to the rise of resistance, health hazards and environmental pollution. Consequently, there is a vital need to develop novel alternatives to those pesticides.

Synthetic AChE inhibitors are often developed through rational drug design, utilizing various heterocyclic scaffolds, such as carbamates, organophosphates, and triazoles, to optimize potency, selectivity, and pharmacokinetics. These compounds can interact with the catalytic active site and peripheral anionic site of the enzyme, improving their inhibitory efficiency. Continued research into synthetic AChE inhibitors aims to develop safer, more selective molecules with reduced side effects and environmental impact^9^.

Benzochromenes, *O*-containing fused heterocyclic compounds, are among the most important molecules in light of their biomedical applications^10^. They are the most worthwhile pharmacological compounds associated with a broad range of significant biological properties, such as antimicrobial^11–14^, antioxidant^15,16^, antiviral^17,18^, anticancer^19,20^ and Alzheimer’s preventative^21^. Moreover, substituted 4*H*-chromenes have played a crucial role in synthetic strategies to promising motifs in the field of medicinal chemistry in particular hypolipidemic^22^, anti-rheumatic^23^, blood platelet anti-aggregating^24^, vascular-disrupting^25^ and analgesic activities^26^. Fused benzochromenes have gotten to be some of the key molecules employed in the synthesis of vigorous antitumor compounds and other medicines of human sicknesses. For example, crolibulin™ is right now in clinical endeavors for the treatment of solid tumors^27^, and LY290181 is utilized for treating the diabetes-induced vascular dysfunction^28^. Another pharmacophore ingredient, pyrimidine, is an important type of heterocyclic compounds with a wide assortment of biological activities. Pyrimidines have acquired substantial consideration due to their function in biological systems, notably in nucleic acids, which contain purines and pyrimidines as the significant nucleobases^29^. It has been reported that the insertion of an additional nucleus to the pyrimidine core leads to exert a profound effect on conferring new biological activities in these molecules^30^.

On the other hand, triazolopyrimidine derivatives, a subtype of purine analogs, have been reported as the topic of chemical and biological studies owing to their motivating pharmacology concerning antihypertensive, cardiac stimulant, analgesic, anti-HBV, antimicrobial, antipyretic, antifungal, anti-inflammatory, antimalarial, leishmanicidal anticancer, and potential herbicidal activities^31–37^. Furthermore, Trapidil, triazolopyrimidine derivative, serves as a phosphodiesterase inhibitor and a platelet-derived growth factor antagonist^31^. The numerous biological activities of these pharmacophores; benzochromenes and triazolopyrimidines, encourage us to carry on our efforts to synthesize biologically active ingredients for the finding of insecticidal agents with new molecular structures.

## Results

### Synthesis of ethyl formimidate derivative 2

**Fig. 1:**
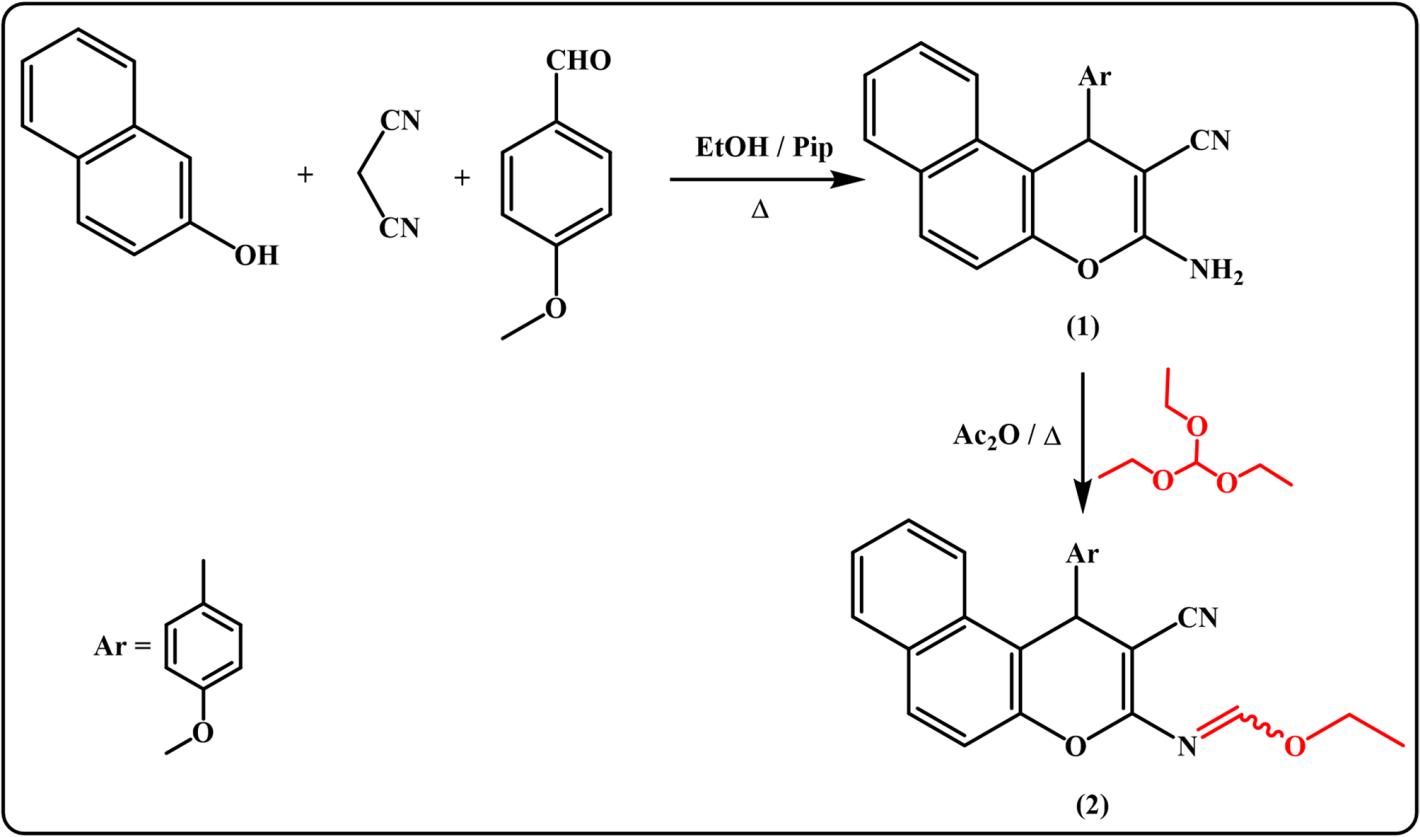
Synthesis of ethyl formimidate derivative 2.

As a part of our endeavors to synthesize oxygen-containing heterocyclic compounds with anticipated biological activity^38–57^, we aimed to develop a new series of benzo[f]chromeno[2,3-d]pyrimidines and/or chromenotriazolo[1,5-c]pyrimidines, and evaluate their insecticidal properties. For this purpose, the key intermediate, benzo[*f*]chromene-2-carbonitrile derivative **1**^58^, was generated through one-pot three component reaction of *β*-naphthol, malononitrile, and *p*-anisaldehyde in absolute ethanol containing a catalytic amount of piperidine. In order to increase the reactivity of the deactivated amino group of the enaminonitrile derivative **1**, it was reacted with triethyl orthoformate in the presence of freshly distilled acetic anhydride, yielding ethyl formimidate derivative **2**, which exists as a Syn/Anti mixture in a 24:76 ratio, as shown in Fig. 1.

### Results of reaction of ethyl formimidate derivative 2 with some heterocyclic amines

**Fig. 2:**
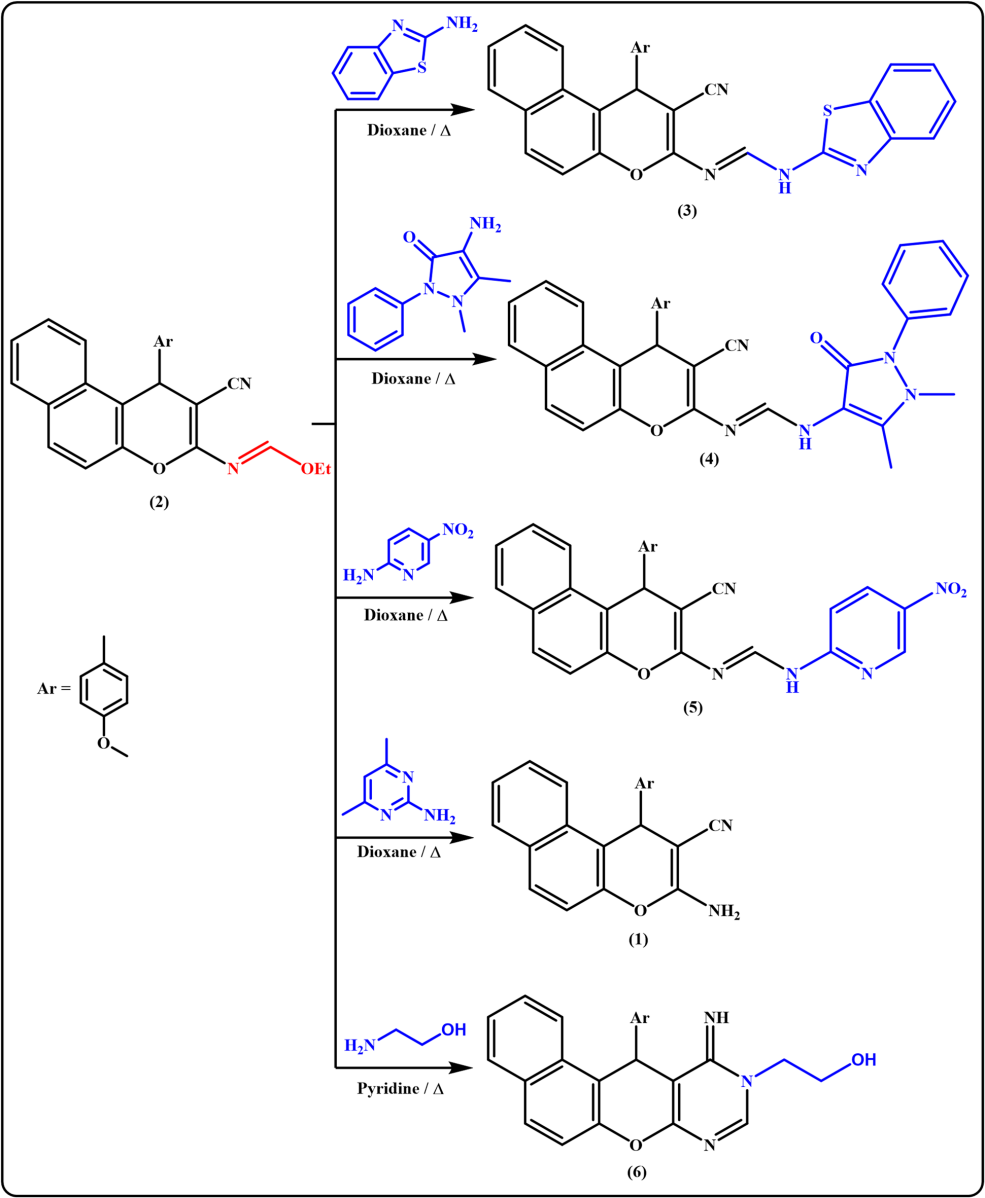
Reaction of ethyl formimidate derivative 2 with some heterocyclic amines.

Initially, ethyl formimidate **2** was subjected to react with various *N*-nucleophiles in an attempt to synthesis new chromenopyrimidine derivatives. Thus, treatment of **2** with different heterocyclic amines such as benzo[*d*]thiazol-2-amine, 4-amino antipyrin and/or 2-amino-5-nitro pyridine in boiling dioxane resulted in the uncyclized formamidine derivative **3**–**5** in yields of 55–75% (Fig. 2). The characteristic absorption bands for C ≡ N at 2209, 2194, and 2213 cm^-1^ in IR spectra of the formamidines **3**–**5** alongside the appearance of exchangeable singlet signals characteristic for NH protons in the ^1^H-NMR spectra reinforce the suggested structures. In contrast, reacting compound **2** with 2-amino-4,6-dimethyl pyrimidine under the same reaction conditions afforded the enaminonitrile derivative **1** in 80% yield instead of the expected pyrimidine derivative. The spectroscopic data are in good agreement with the proposed structure. Aminolysis of **2** using ethanol amine in boiling pyridine furnished the chromenopyrimidine derivative **6** as white crystals in 60% yield which was supported from the IR spectrum that devoid any absorption band for the CN function. Also, the ^1^H-NMR and ^13^C-NMR spectra were in accord with the assigned structure 6.

### Results of reaction of ethyl formimidate derivative 2 with some acid Hydrazides

**Fig. 3:**
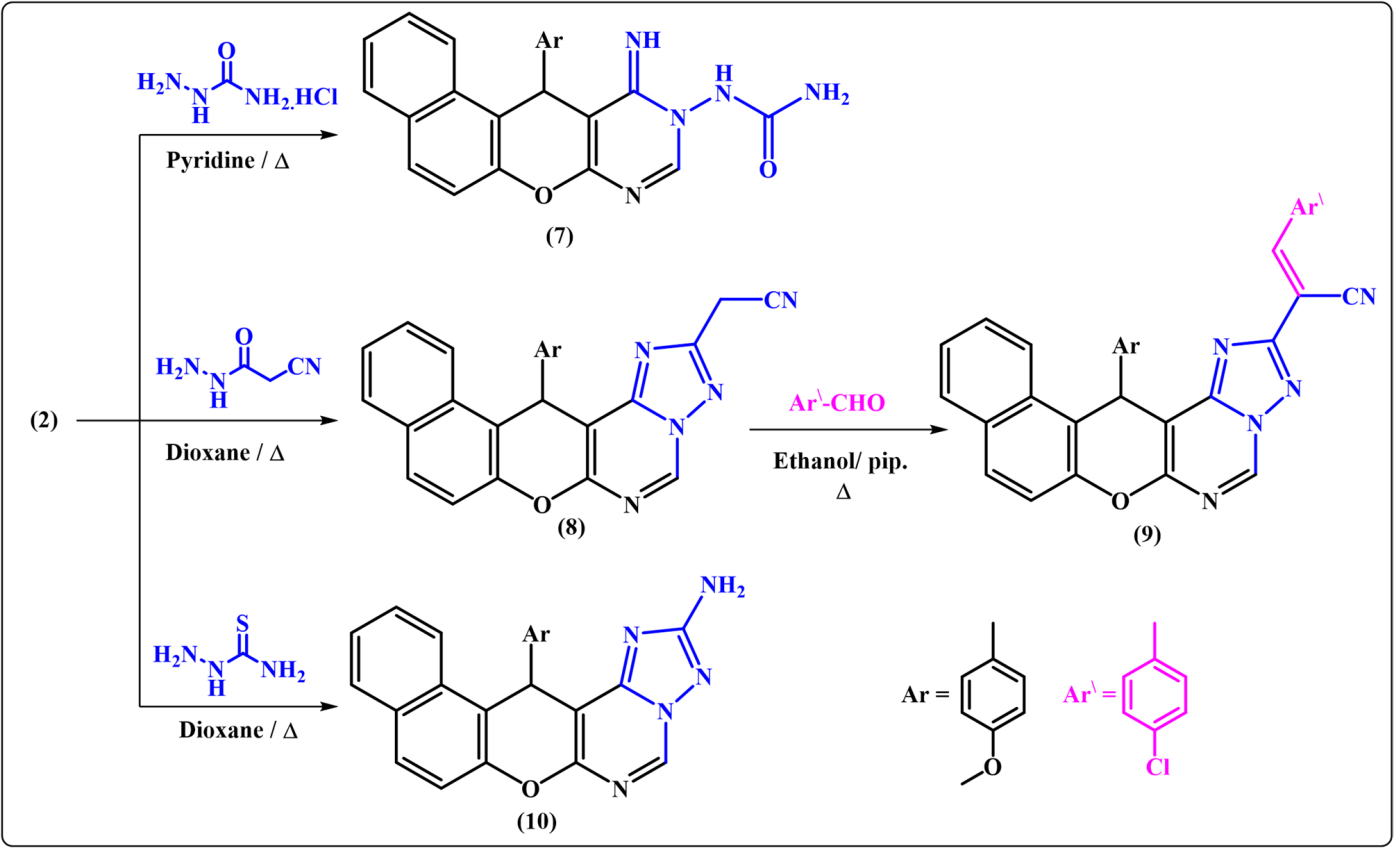
Reaction of ethyl formimidate derivative 2 with some acid hydrazides.

In this study, the reactivity of **2** towards some acid hydrazides namely, semicarbazide hydrochloride, cyanoacetohydrazide, and thiosemicarbazide has been investigated as illustrated in Fig. 3. Meanwhile, the triheterocyclic compound **2** was established as a mediator for construction of tetra- and pentaheterocyclic compounds. Thus, when the key intermediate, ethyl formimidate derivative **2**, was subjected to react with semicarbazide hydrochloride in boiling pyridine, the corresponding benzochromenopyrimidine **7** was obtained as a sole product in 80% yield.

In our study, the synthesis of polyfunctionalized binary and fused triazole scaffolds allowing for the efficient introduction of functional groups that are known to enhance bioactivity. These scaffolds were specifically designed based on SAR insights and prior literature demonstrating the insecticidal potential of 1,2,4-triazoles and related heterocycles^59^.

Acetonitrile derivative **8** was generated as yellow solid in 55% yield through reaction of **2** with 2-cyanoacetohydrazide in refluxing dioxane (Scheme 3). The structure of compound **8** was suggested from studying analytical and spectral data. For instance, the IR spectrum showed υC ≡ N at 2255 cm^-1^ (saturated nitrile) as well as ^1^H NMR spectrum exhibited the appearance of a singlet signal attributable to the –CH_2_CN protons at δ 4.49 ppm and thus proved the result of the cyclization reaction.

Notably, base-catalyzed condensation of cyanomethyl derivatives with aromatic aldehydes gave Knoevenagel products. Similarly, when cyanomethyl derivative **8** was allowed to react with *p*-chlorobenzaldehyde in boiling dioxane containing a catalytic amount of piperidine, the desired arylidene derivative **9** was isolated as a sole product. Ultimately, amino triazolopyrimidine derivative **10** was also obtained as brown crystals in 70% yield from heating ethyl formamidate **2** with thiosemicarbazide in dioxane under reflux. The structure of **10** was elucidated by elemental analysis and spectroscopic data.

### Theoretical investigations

#### Molecular orbital calculations

**Fig. 4:**
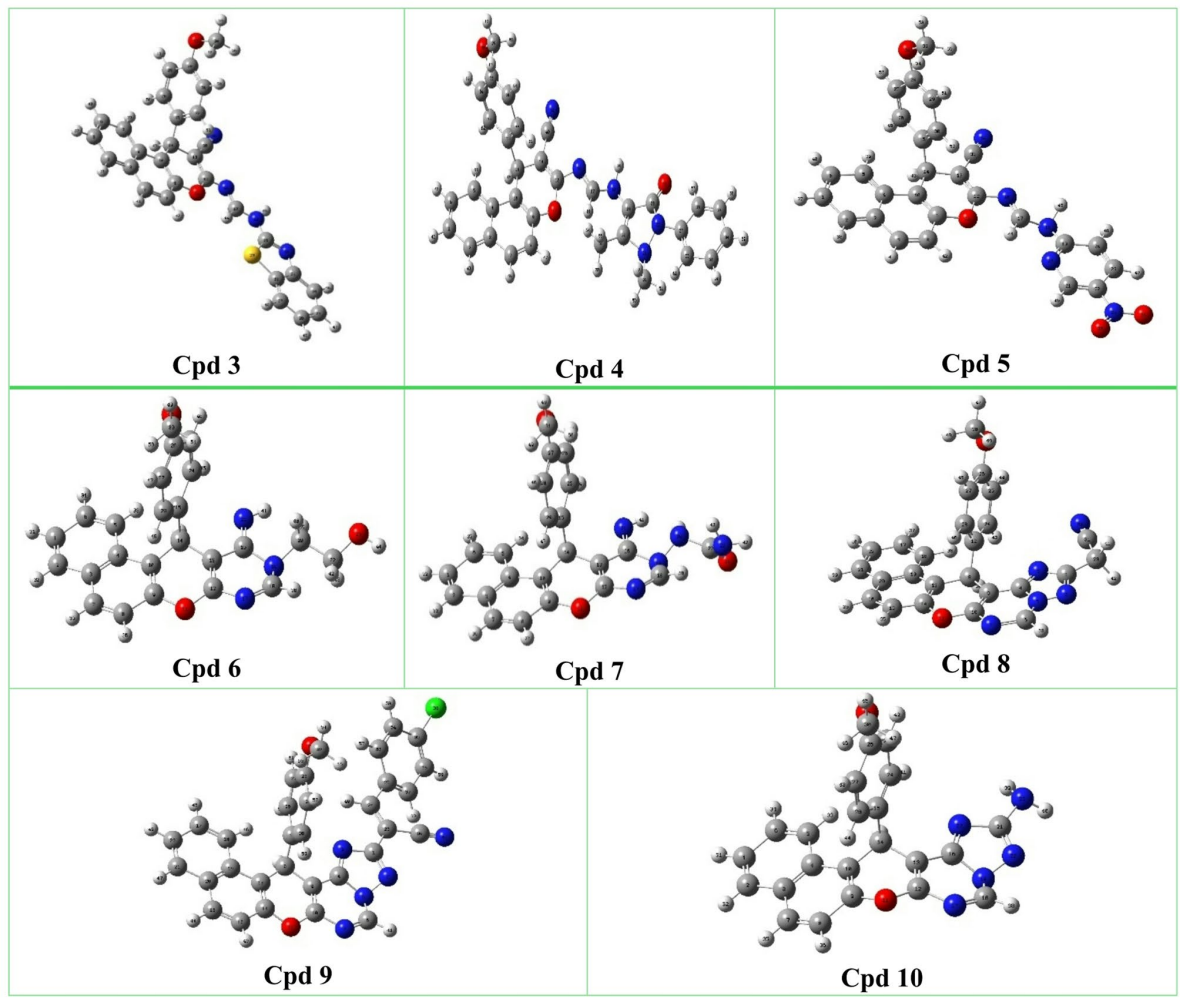
Optimized structures for the synthesized compounds using the 6–31G(d) basis set.

Understanding chemical reactivity is a crucial step that can be assessed through various quantum chemical descriptors. This analysis is carried out using Density Functional Theory (DFT), which provides detailed insights into a molecule’s electronic structure through the frontier molecular orbital (FMO) method. In this approach, key parameters such as the highest occupied molecular orbital (HOMO), the lowest unoccupied molecular orbital (LUMO), and related properties are examined to better understand intermolecular interactions. The frontier molecular orbitals of the synthesized compounds were calculated using the B3LYP/6–311 + + G(d, p) basis set for the optimization of the synthesized compounds^60^. The fully optimized molecular geometries, along with their atom numbering planner, are presented in (Fig. 4). The optimized energies of the compounds range from (-822485.62) kcal/mol to (-1182642.00) kcal/mol. Among them, compound **10** exhibited the highest stability, indicated by the lowest total energy. In contrast, compound **3** showed the lowest stability, reflected by its highest total energy. Theoretical calculations provide insight into the electron distribution of the HOMO and LUMO energy levels of the synthesized derivatives. In compounds **3** and **4**, the HOMO electrons are mainly localized on the benzothiazole and antipyrinemoieties, respectively, whereas the LUMO shows a notable relocation of electron density toward the chromene ring. In contrast, for compound **5**, the HOMO electron density is spread over the benzochromene ring, while in the LUMO, it completely shifted toward the pyridine core.

**Fig. 5:**
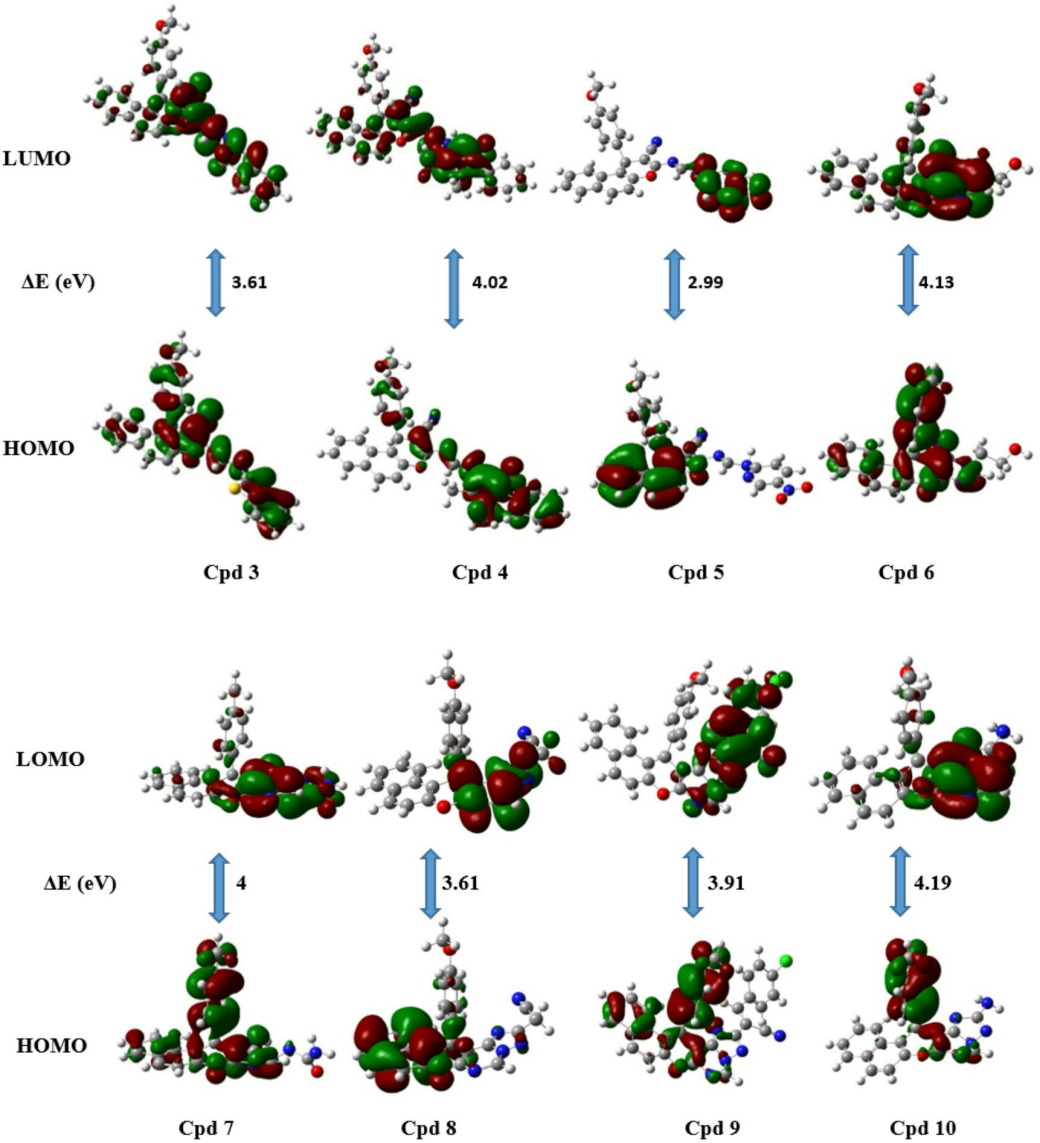
Frontier molecular orbitals of the synthesized compounds computed by the B3LYP/6-31G (d) method.

Similar findings were observed for compounds **6–10**, In the HOMO, the electron density is primarily distributed over the pyrimidine and anisole moieties in compounds **6**, **7**, **9**, and **10**, and over the benzochromene ring in compound **8**. For the LUMO, the electron density shifts to the triazolopyrimidine ring in compounds **8**–**10**, while in compounds **6** and **7**, it relocates to the pyrimidine ring. Consequently, a considerable HOMO-LUMO energy gap (ΔEgap) is observed in the conjugated system, likely due to charge transfer between the electron-donating and electron-withdrawing groups through the π bridge (Fig. 5). As depicted in Table 1, the energy gaps between HOMO and LUMO are found in the range from 2.99 to 4.38 eV. Derivative **5** (2.99 eV), along with derivatives **3** and **8** (3.61 eV), exhibit the lowest energy gaps (ΔEgap), suggesting higher polarizability, increased chemical reactivity, and reduced kinetic stability. Based on the HOMO and LUMO values, several parameters were calculated, including ionization potential (I), electron affinity (A), chemical hardness (ɳ), softness (s), chemical potential (µ), electrophilicity index (ω), and electronegativity (χ). It was observed that derivatives with smaller energy gaps exhibit lower hardness, making them chemically more reactive, while the inverse was found in the case of softness. Additionally, a higher ionization potential (*I*_P_) and a negative value of chemical potential (µ) contribute to assessing the stability of the derivatives. Nevertheless, the high electron affinity (A) and electronegativity (χ) indicate that the derivatives have a strong ability to attract electrons, facilitating charge transfer and thereby enhancing electrical conductivity. Moreover, the electrophilicity index (ω) is used to determine the global electrophilicity of the derivatives, offering valuable insights into their potential biological activity. For instance, compound **5** exhibited a relatively high ω value of 6.67 eV, suggesting strong electrophilic character, whereas compound **6** showed a significantly lower ω value of 3.12 eV. Additional quantum chemical parameters are presented in Table 1.

**Table 1:** Energy (eV) of HOMO, LUMO, energy gap (ΔE), hardness (η), softness (S), chemical potential (µ), electrophilicity index (ω), electronic affinity (A), ionization potential (I_*P*_), and electronegativity (χ) of derivatives **3–10**.

Cpd	E_T_ (kcal/mol)	E_HOMO_ (eV)	E_LUMO_ (eV)	ΔE (eV)	µ (eV)	η (eV)	S (eV^− 1^)	ω (eV)	A (eV)	I_*P*_ (eV)	χ (eV)
**3**	-1182642.00	-5.523	-1.904	3.61	-3.71	1.80	0.55	3.82	1.904	5.523	3.71
**4**	-1112417.42	-5.687	-1.659	4.02	-3.67	2.01	0.49	3.35	1.659	5.687	3.67
**5**	-1013187.02	-5.959	-2.966	2.99	-4.46	1.49	0.67	6.67	2.966	5.959	4.46
**6**	-826386.05	-5.659	-1.523	4.13	-3.59	2.06	0.48	3.12	1.523	5.659	3.59
**7**	-870412.11	-5.741	-1.741	4	-3.74	2	0.5	3.49	1.741	5.741	3.74
**8**	-870280.33	-5.578	-1.959	3.61	-3.76	1.80	0.55	3.92	1.959	5.578	3.76
**9**	-1327540.21	-6.013	-2.095	3.91	-4.05	1.95	0.51	4.20	2.095	6.013	4.05
**10**	-822482.94	-5.823	-1.632	4.19	-3.72	2.09	0.47	3.31	1.632	5.823	3.72

#### Molecular electrostatic potential (MEP) surface

**Fig. 6:**
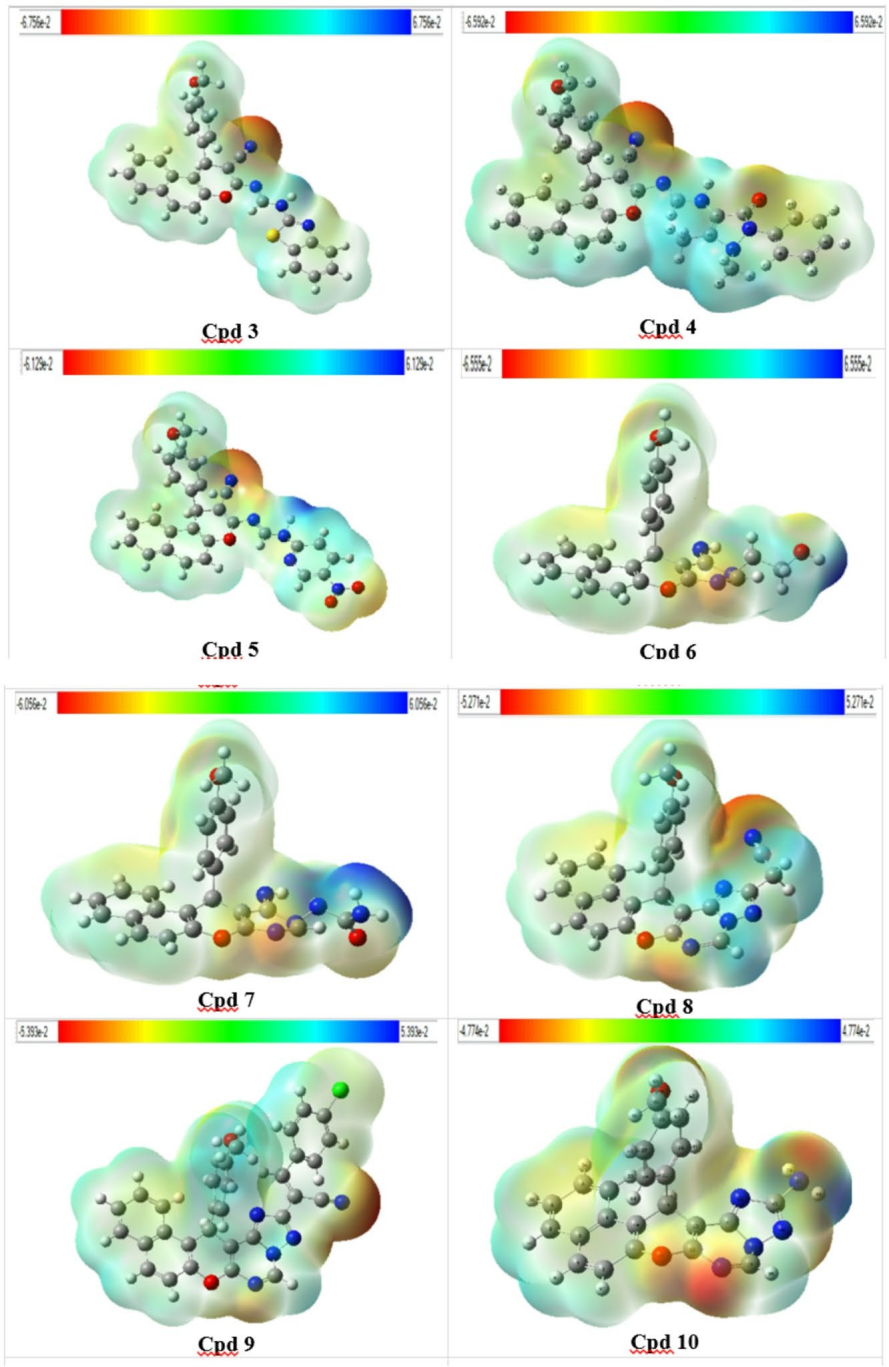
Molecular electrostatic potential (MEP) maps of the prepared compounds.

The Molecular Electrostatic Potential (MEP) serves as a valuable theoretical approach for analyzing molecular structures, assessing physicochemical properties, and predicting chemical reactivity. In this study, MEP was computed using the DFT/B3LYP/6–311 + + G(d, p) method, as shown in Fig. 6. Different colors represent varying electrostatic potential values on the molecular surface. Regions shown in blue indicate areas of positive potential and are typically considered electrophilic centers, while red regions represent negative potential, corresponding to nucleophilic sites. Green areas reflect regions with minimal electrostatic potential. In the MEP map, positive potential is mainly observed over carbon and hydrogen atoms (blue), whereas negative potential is primarily localized around nitrogen and oxygen atoms, depicted in red and yellow colors.

### Biological activity

Synthesized compounds were tested against *C. pipiens* larvae and adults. The majority of the tested compounds showed good activity, especially against adults in comparison to reference insecticides recommended by WHO.

#### Larvicidal bioassay activity

The compounds **10** and **5** showed strong potency, more than temephos, against larvae with LC_50_s of 40.15 and 46.17 ppm, respectively. While **3**, **4** and **7** were approximately equivalent to temephos against larvae with LC_50_s of 56.20, 69.13, 83.65 and 59.83 ppm, respectively, as shown in Table 2. On the other hand, some compounds showed low activity as **8**, **9** and **6**, which exhibited 113.78, 144.56 and 215.40 ppm, respectively. Therefore, the compounds were arranged according to their potency as follows: 10 > 5 > 3 > temephos > 4 > 7 > 8 > 9, where they were more effective than **6** with 5.36, 4.66, 3.83, 3.60, 3.11, 2.57, 1.89 and 1.49 folds. It was noted that the larval population showed a heterogeneous response to 4 and temephos due to the high chi-square values. (Supplementary file)

**Table 2:** Larvicidal toxicity parameters of benzochromene derivatives against early third instar larvae of *Culex pipiens* at 48 h post-treatment.

Compd. (ppm)	LC_25_ (^*^F.l. at 95%)	LC_50_ (^*^F.l. at 95%)	LC_90_ (^*^F.l. at 95%)	^**^Slope ± SE	^***^X^2^	Relative potency
**3**	16.08 (9.33–23.37)	56.20 (42.61–69.97)	605.11 (425.93- 1005.42)	1.24 ± 0.13	6.26	3.83
**4**	18.33 (10.55–26.66)	69.13 (53.07–85.88)	860.87 (571.40- 1584.50)	1.17 ± 0.12	6.74	3.11
**5**	17.01 (5.92–23.43)	46.17 (24.22–64.92)	307.82 (223.80- 706.26)	1.55 ± 0.14	10.97	4.66
**6**	52.17 (35.48–68.55)	215.40 (171.30- 286.41)	3186.15 (1683.67- 8776.88)	1.09 ± 0.13	3.88	1
**7**	22.27 (13.40- 31.54)	83.65 (65.79- 103.05)	1033.81 (671.83- 1967.07)	1.17 ± 0.12	5.01	2.57
**8**	28.12 (17.16–39.33)	113.78 (90.71- 141.64)	1619.89 (967.39- 3583.61)	1.11 ± 0.12	4.59	1.89
**9**	36.86 (24.00- 49.69)	144.56 (116.83- 181.32)	1939.30 (1136.94- 4407.57)	1.13 ± 0.12	2.72	1.49
**10**	13.04 (7.53- 19.00)	40.15 (29.72–50.50)	340.02 (253.86- 513.38)	1.38 ± 0.14	4.84	5.36
**Temephos**	15.96 (2.86–21.25)	59.83 (25.12–91.06)	736.30 (553.04- 4075.96)	1.17 ± 0.12	11.55	3.60

*F.l.: Fiducial limits; **Slope ± SE: slope of concentration–mortality regression line ± standard error; **χ²**: chi-square at significance value (< 0.05); Relative potency calculated relative to compound 6.

#### Adulticidal bioassay activity

The dose-dependent study was performed to assess the LC_50_ values of the tested compounds relative to deltamethrin. Although all tested compounds were more toxic than deltamethrin, **6** were slightly active. Each of **10**, **5**, **3**, **4**, **7**, **8**, **9**, and **6** were 5.28, 4.44, 3.54, 2.79, 2.15, 1.58, 1.28 and 1.07 times more effective than deltamethrin, respectively, as shown in Table 3. At the same manner of larvicidal activity, **10** and **5** exerted high potencies with LC_50_ values of 55.02 and 65.43 ppm, respectively. Moderate activity against adults was achieved by **3** (82.11 ppm), **4** (104.07 ppm) and **7** (135.07 ppm). The least adulticidal activity was exhibited by **8**, **9** and **6** with LC_50_s of 182.96, 225.69 and 270.83 ppm, respectively. Conversely to larvicidal activity, the adults showed a homogenous response to all tested compounds. (Supplementary file)

**Table 3:** Adulticidal toxicity parameters of benzochromene derivatives against mixed sex adults of *Culex pipiens* at 48 h post-treatment.

Compd. (ppm)	LC_25_ (^*^F.l. at 95%)	LC_50_ (^*^F.l. at 95%)	LC_90_ (^*^F.l. at 95%)	^**^Slope ± SE	^***^X^2^	Relative potency
**3**	22.83 (14.08–31.93)	82.11 (64.97- 100.59)	933.99 (622.33- 1697.36)	1.21± 0.12	1.99	3.54
**4**	27.58 (17.28–38.11)	104.07 (83.40- 128.02)	1297.45 (817.19- 2599.34)	1.16± 0.12	3.31	2.79
**5**	19.22 (11.74–27.11)	65.43 (50.99–80.35)	670.66 (470.51- 1116.63)	1.26± 0.13	3.76	4.44
**6**	93.87 (74.87- 112.96	270.83 (224.00- 343.10)	2027.94 (1283.92- 3963.66)	1.46± 0.15	1.77	1.07
**7**	38.14 (25.93–50.27)	135.07 (110.64- 165.87)	1492.78 (939.43- 2971.85)	1.22± 0.13	1.66	2.15
**8**	50.72 (35.80- 65.36)	182.96 (149.37- 231.08)	2094.23 (1243.13- 4615.20)	1.21± 0.13	0.79	1.58
**9**	69.45 (52.52–86.13)	225.69 (185.44- 286.28)	2118.48 (1292.34- 4422.79)	1.31± 0.14	0.60	1.28
**10**	16.08 (9.43–23.25)	55.02 (41.79–68.39)	569.32 (405.26- 927.39)	1.26 ± 0.13	3.50	5.28
**Deltamethrin**	112.18 (92.20- 132.52)	290.83 (243.47- 362.82)	1777.08 (1178.57- 3214.80)	1.63 ± 0.16	1.59	1

*F.l.: Fiducial limits; **Slope ± SE: slope of concentration–mortality regression line ± standard error; **χ²**: chi-square at significance value (< 0.05); Relative potency calculated relative to deltamethrin.

### Cytotoxicity

To assess the safety of the most potent insecticidal derivatives, compounds **5** and **10** were subjected to in vitro cytotoxicity evaluation using the WI-38 human lung fibroblast cell line, which represents non-cancerous, healthy human tissue. The cytotoxicity was evaluated *via* the MTT assay, using doxorubicin as a positive control. Compound **5** exhibited an IC₅₀ value of 72.65 ± 10.8 µM, while compound **10** showed an IC₅₀ of 90.32 ± 5.6 µM. These values indicate low cytotoxicity toward normal human cells and suggest a favorable selectivity index. The results support the conclusion that compounds **5** and **10** not only demonstrate strong insecticidal efficacy but also maintain a promising safety profile for potential development as insecticidal agents. These findings confirm the initial hypothesis of the study that the synthesized compounds act as AChE inhibitors with low toxicity to humans, likely due to differences in target site structure and interaction. (Table 4)

**Table 4:** Cytotoxicity of tested compounds against WI-38 cell line.

Compound	In vitro Cytotoxicity IC_50_ (µM)* WI-38
**5**	72.65 ± 10.8
**10**	90.32 ± 5.6
**Doxorubicin**	6.71 ± 0.5

* IC_50_ (µM): 1–10 (very strong), 11–20 (strong), 21–50 (moderate), 51–100 (weak) and above 100 (non-cytotoxic).

### Molecular docking

**Fig. 7:**
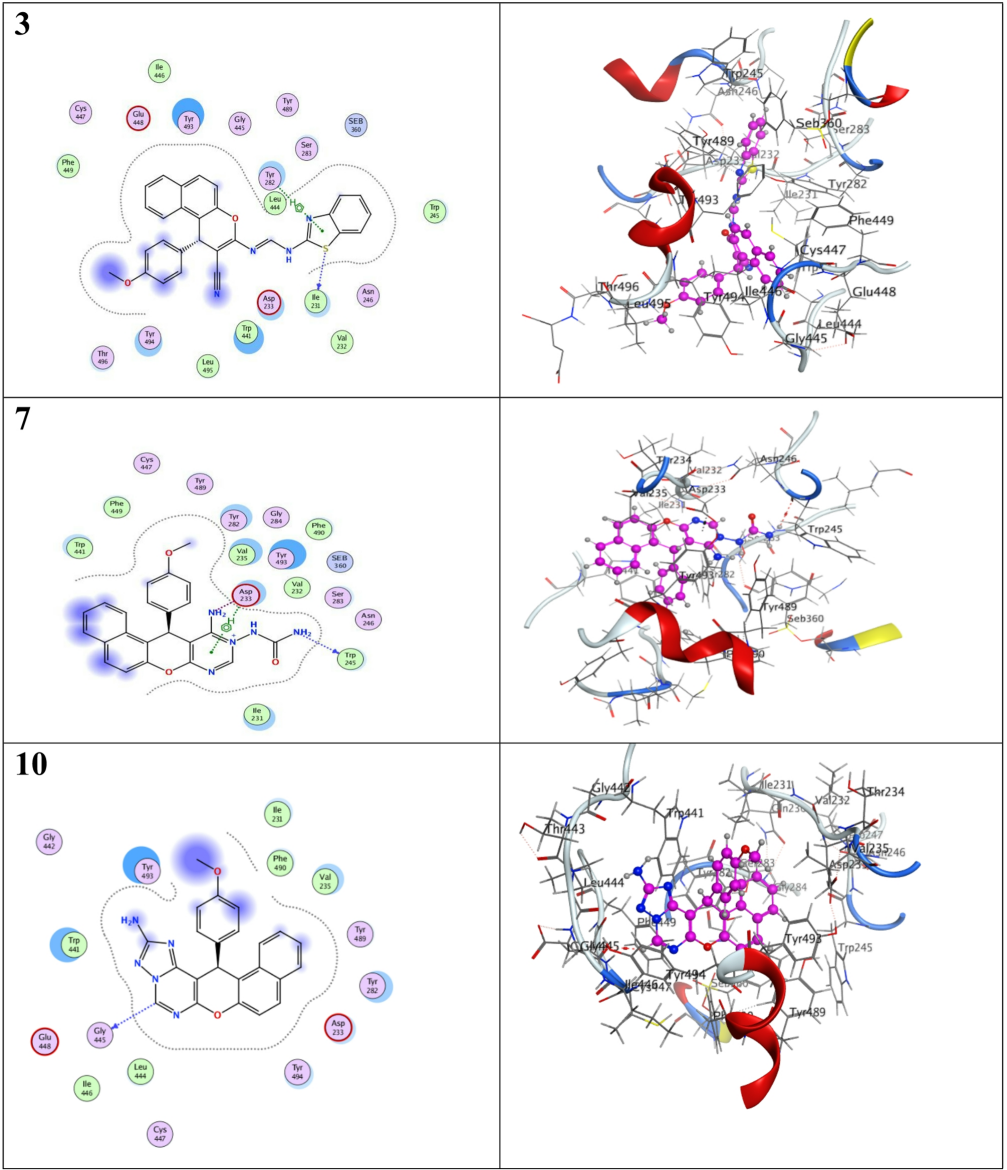

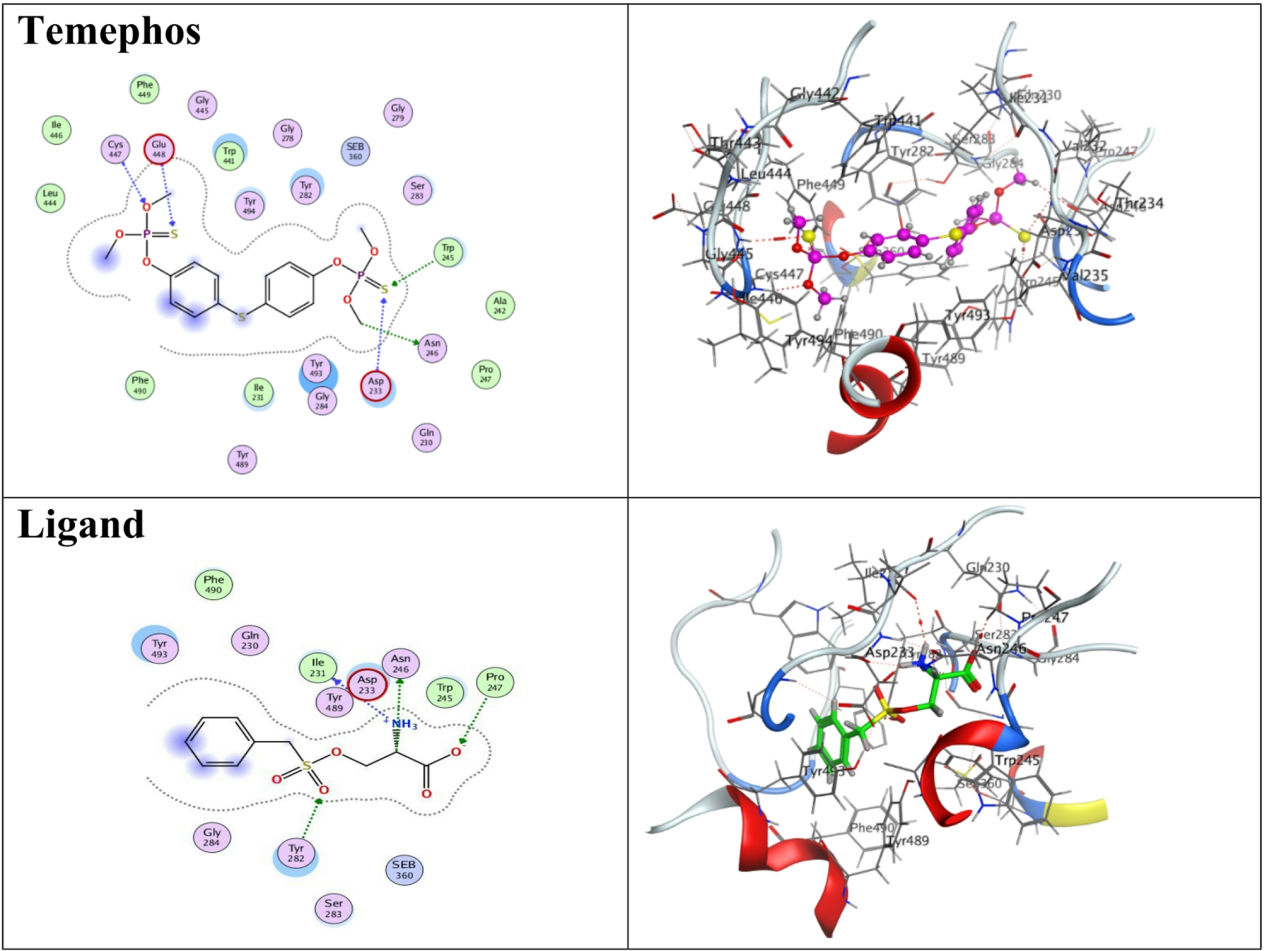
2D and 3D interaction visualization of tested compounds, temephos and ligand with the target site of *Anopheles gambiae* AChE (PDB code: 5YDJ).

The tested compounds, ligand and reference organophosphate insecticide, temephos, were docked to the target enzyme, *Anopheles gambiae* acetylcholinesterase (AChE), to study their binding modes. The binding affinities and types of interactions of the tested compounds to *A. gambiae* AChE (PDB code: 5YDJ) were configured to illustrate their mode of action. The docking parameters, including the type of interaction and binding energies of the docked compounds, were demonstrated in Table 5. The AChE inhibitor, temephos, formed five hydrogen bonds with the target site amino acids, Asn 246, Asp 233, Trp 245, Glu 448 and Cys 447, in addition to exerting a high docking energy of − 8.11 kcal/mol (Fig. 4). Compound **3** exhibited two different bonds, a H-bond with Ile 231 and Pi-H with Tyr 282 as visualized in Fig. 6 and showed a docking score of -7.565 kcal/mol (Table 5). While compound **7** interacted with Trp 245 through H-bonding as well as ionic and pi-H with Asp 233 of the AChE active site, so exerted binding energy of -7.330 kcal/mol. On the converse, compound **10** showed only H-bonding with Gly 445 and a low interaction energy of -6.51 kcal/mol. The ligand was redocked to the target site, forming four hydrogen bonds with Ile 231, Asn 246, Pro 247, and Tyr 282, but showed a low binding affinity of -5.40 kcal/mol. All compound alignments, binding interactions and 2D and 3D structures are shown in Fig. 7.

**Table 5:** Molecular Docking scores and interaction profiles of the synthesized candidates with *Anopheles Gambiae* acetylcholinesterase (AChE).

Cpd	Docking score (kcal/mol)	rmsd_refine	Binding site		Type of interaction	Interaction Distance	E (kcal/mol)
			Ligand	Receptor			
**3**	-7.565	1.855	S (32) 5-ring	ILE 231 (A) TYR 282 (A)	H-donor Pi-H	3.22 3.82	0 -1.1
**4**	-7.844	1.717	5-ring	TYR 282 (A)	pi-H	3.66	-2.0
**5**	-7.702	1.240	6-ring	TRP 441 (A)	H-pi	4.16	-0.8
**6**	-7.360	1.345	O (37) 6-ring	ILE 231 (A) ASP 233 (A)	H-donor pi-H	2.78 3.90	-1.8 -0.7
**7**	-7.330	1.260	N (34) N (30) 6-ring	TRP 245 (A) ASP 233 (A) ASP 233 (A)	H-donor ionic pi-H	3.26 3.86 3.43	-0.9 -0.8 -0.8
**8**	-7.155	1.219	N (49) 6-ring 5-ring	TYR 282 (A) TYR 493 (A) TYR 493 (A)	H-acceptor pi-H pi-pi	3.29 4.44 3.88	-1.1 -0.8 0
**9**	-7.429	1.636	CL (44)	GLN 230 (A)	H-donor	3.50	-0.4
**10**	-6.513	1.367	C (18)	GLY 445 (A)	H-donor	3.24	-1.2
**Temephos**	-8.116	1.603	C (1) S (70 S (7) S (37) O (38)	ASN 246 (A) ASP 233 (A) TRP 245 (A) GLU 448 (A) CYS 447 (A)	H-donor H-acceptor H-acceptor H-acceptor H-acceptor	3.46 4.42 3.55 3.89 3.36	-0.7 -1.2 -1.0 -3.1 -0.6
**Redocked ligand**	-5.407	1.381	N (2) C (6) O (12) O (16)	ILE 231 (A) ASN 246 (A) PRO 247 (A) TYR 282 (A)	H-donor H-donor H-acceptor H-acceptor	3.60 3.48 3.43 2.59	-0.8 -0.8 -0.7 -2.4

## Discussion

Chromene is a natural compound frequently found in nature and is a widely used scaffold for designing therapeutic and diagnostic chemicals. It is a heterocyclic system that consists of an oxine ring fused to a phenyl ring, which occurs in various natural compounds^61^. Precocene-I (7-methoxy-2,2-dimethyl-2 H-chromene) and precocene-II (6,7-dimethoxy-2,2-dimethyl-2 H-chromene) are chromene-based botanical compounds extracted from the weed, *Ageratum houstonianum* that act as insect growth regulators (IGR) and acetylcholinesterase (AChE) inhibitors^6^. Some synthetic chroman and chromene analogs showed moderate to good potencies against susceptible and permethrin-resistant strains of *Aedes aegypti* larvae and adults^62^. Chromene amide analogs were synthesized based on a natural chromene amide extracted from *Amyris texana*. These compounds showed significant mortalities against Formosan subterranean termites^63^.

The activity of synthesized benzochromenes differs in adults and larvae; they were more active against larvae than adults. This may be due to the time of exposure, the method of application, or the habitat and behavior of each stage. Chromenes with aldehydes or alcohol substitutions showed moderate toxicity when applied by the microinjection method, while similar compounds showed high activity when ingested. Moreover, these scaffolds significantly increased the developmental malformations; therefore, they act as growth regulators for *Spodoptera frugiperda*^64^. The chromen-based compounds had toxic and biological impacts against *Pectinophora gossypiella*,* Earias insulana* and *Helicoverpa armigera.* Newly hatched larvae of *H. armigera* showed high mortalities, followed by *E. insulana* and *P. gossypiella*, after treatment with chromen compounds. Moreover, these compounds had latent effects, including accumulated larval and pupal mortalities, a significant decrease in larval and pupal weight, and a reduction in fecundity^65^.

Compounds 10 and 5 with pyrimidine and pyridine moieties, respectively, showed high toxicity, which aligns with the previous results, which showed insecticidal efficiency against both nymphs and adults of cowpea aphid^66^. Pyridine derivatives of 3-(Substituted) methylthio-5, 6, 7, 8-tetrahydroisoquinoline-4-carbonitriles exhibited good insecticidal potencies against *Aphis gossypii* and their mode of action was elucidated by a molecular docking study^67^. Some derivatives, acyl hydrazides, were assessed for their insecticidal and biochemical effectiveness against second and fourth-instar larvae of the *Spodoptera littoralis*. The treatment with the LC_50_ of the tested compounds affected the activity of various enzymes involved in the cuticle production of *S. littoralis* larvae in their fourth instar^68^.

Molecular docking study indicated that temephos showed high affinity to bind to AChE pocket and form strong H-bonds relative to other tested compounds. Temephos is an organophosphate insecticide and is classified in the Insecticide Resistance Action Committee (IRAC) as AChE inhibitor (1B)^69^. Although compound **10** showed high insecticidal activity against larvae and adults of *C. pipiens*, it showed low interaction affinity and docking energy to AChE. Compound **10** contains triazole and pyrimidine rings fused to benzochromene, where triazole targets the GABA receptor^70^, and pyrimidine disrupts the normal development and growth of *C. pipiens*^71^.

## Structure-activity relationship

The tested compounds contain a common benzochromene skeleton bearing a cyano group and different heterocyclic or amine substituents. There are great variations in larvicidal and adulticidal activities based on the substitutions inserted. Aminotriazolopyrimidine derivative **10** was the most effective compound because of the formation of pyrimidine and triazole rings, where pyrimidine and aminotriazole derivatives had insecticidal activity against *C. pipiens* and *Plodia interpunctella*, respectively^71^. Amino triazole disrupts the nervous system function of insects by binding to GABA receptors^72^. The amino group attached to triazole is electron-donating, a small bulking that increases electron density on the triazole ring, therefore strengthening electrostatic or π–π interactions with aromatic amino acids in the target sites. On contrast, cyano methyl group linked to triazole in compounds **8** and **9** is electron-withdrawing group and more bulking, which withdraws electrons from the ring, reducing nucleophilicity and binding affinity toward the active site. Moreover, the bulking of **9** decreased its insecticidal toxicity than **8**^73^.

From scheme 2, it was noted that the direct attachment of the cyano group (electron-withdrawing group) to benzochromene influenced the activity of synthesized compounds, which increases the resonance and enhances hydrophobic interaction with the target site. Additionally, the toxicity of compound **5** increased as a result of pyridine insertion, as pyridine exhibited larvicidal activity against *C. pipiens*^74^. Compound **3** showed good potency might be due to the presence of benzothiazole, which had insecticidal activities against *Aphis fabae* and *Plutella xylostella*^75^. Benzothiazole was investigated for its insecticidal activity against all developmental stages of *Tribolium castaneum*, and its effective repellency against *T. castaneum* in addition to decreasing progeny production^76^. Benzothiazole is suggested to be an inhibitor of *Anopheles gambiae* and *A. funestus* trehalase; therefore, the insect fails to adapt to flight and stress^77^. Benzothiazole compounds stimulate the release of calcium ions from the central neurons of insects, which disrupt calcium homeostasis within neurons, ultimately causing insecticidal effects^78^. The 4-amino-1,5-dimethyl-2-phenyl pyrazolone group influenced the potency of compound **4**, whereas the 5-aminopyrazolo-pyrazolone caused disrupted metamorphosis and deleterious histological malformation in the midgut of *Spodoptera littoralis* larvae. In addition to the phenyl group at position 3, which leads to an increased lipophilicity and allows hydrophobic interactions or π–π stacking with insect target enzymes^79^. In compound **7**, formation of 1-(6-iminopyrimidin-1(6 H)-yl)urea moiety conjugated to benzochromene enhanced the potency, possibly because of the presence pyrimidine ring and urea group. Pyrimidine derivatives exhibited larvicidal activity by mimicking the ecdysone hormone of insects, leading to premature molting and death in insects^70,80^. In conclusion, the presence of different functional groups attached to benzochromene greatly affected the compound’s insecticidal toxicity.

Ultimately, A comparative analysis of the molecular docking results with the biological activity data (larvicidal and adulticidal) reveals a notable degree of concordance, thereby supporting the predictive utility of the in silico approach. Compounds 10 and 5, which exhibited the strongest binding affinities to Anopheles gambiae AChE (-6.51 and − 7.70 kcal/mol, respectively), also demonstrated the highest insecticidal potency in both larval and adult bioassays. These compounds formed stable interactions with key active site residues, such as Gly445, Asp233, and Trp245, which are critical for enzyme inhibition. Their enhanced activity may also be attributed to the electron-donating and planarity-inducing nature of their heterocyclic substituents (e.g., aminotriazolopyrimidine and pyridine), as supported by SAR analysis.

Similarly, compound 3, which formed moderate hydrogen bonding and Pi-H interactions in the docking model, showed comparable bioactivity (LC₅₀ = 56.20 ppm in larvae), further reinforcing the correlation. Conversely, compounds such as 6 and 9, which lacked significant interactions in the docking simulations and showed weaker binding energies, exhibited correspondingly low biological efficacy. Overall, the good alignment between computational predictions and experimental results validates the relevance of the molecular docking, SAR analysis, and biological data, providing a reliable framework for the rational design and optimization of future insecticidal agents.

## Conclusion

In summary, we designed, synthesized, and evaluated a novel series of benzochromene derivatives for their insecticidal activity against both larvae and adult stages of *Culex pipiens* L. The bioassay results revealed that several compounds exhibited potent activity, suggesting their potential as effective agents in vector control strategies. Structure–activity relationship (SAR) analysis provided key insights into the molecular features influencing bioactivity, highlighting the importance of specific substituents on the benzochromene core.

To further rationalize the biological activity, Density Functional Theory (DFT) calculations and Molecular Electrostatic Potential (MEP) mapping were employed. These computational studies provided valuable information on the electronic distribution and reactivity patterns of the molecules, correlating well with the observed insecticidal profiles. Notably, active compounds showed distinctive electronic characteristics and charge distributions that may contribute to enhanced interaction with biological targets. These findings collectively support the benzochromene scaffold as a promising framework for the development of new insecticidal agents.

## Materials and methods

All melting points were measured on a Griffin and George melting-point apparatus (Griffin & Georgy Ltd., Wembley, Middlesex, UK). IR spectra were recorded on Pye Unicam SP1200 spectrophotometer (Pye Unicam Ltd., Cambridge, UK) by using the KBr wafer technique. ^1^H-NMR spectra were determined on a Varian Gemini 300 and 400 MHz on Bruker Avance III using tetramethylsilane as an internal standard (chemical shifts in δ scale), while ^13^C NMR spectra were run at 75 and 100 MHz. Elemental analyses were carried out at the Microanalytical Unit, Faculty of Science, Ain Shams University, using a Perkin-Elmer 2400 CHN elemental analyzer (Waltham, MA), and satisfactory analytical data (± 0.4) were obtained for all compounds. The homogeneity of the synthesized compounds was controlled by thin layer chromatography (TLC), using aluminum sheet silica gel F_254_ (Merck).

### 3-amino-1-(4-methoxyphenyl)-1H-benzo[f]chromene-2-carbonitrile 1

A solution of malononitrile (0.66 g, 10 mmol), *p*-anisaldehyde (1.36 mL, 10 mmol), and *β*-naphthol (1.44 g, 10 mmol) in absolute ethanol (30 mL) containing a catalytic amount of piperidine (0.5 mL) was refluxed for 5 h. The deposited solid while heating was filtered off, dried and then crystallized from benzene to give **1** as beige crystals; yield 90%; mp 190–192 °C; IR (KBr, ν, cm^− 1^): 3433, 3343 (NH_2_), 3071 (CH, aromatic), 2963, 2927 (CH, aliphatic), 2187 (C ≡ N), 1648 (C = N). ^1^H NMR (300 MHz, DMSO-*d*_6_) δ_ppm_: 3.67 (s, 3 H, OCH_3_), 5.25 (s, 1H, C_4_H-pyran), 6.81 (d, 2 H, Ar-H, *J* = 8.6 Hz), 6.94 (brs, 2 H, NH_2_, exchangeable with D_2_O), 7.11 (d, 2 H, Ar-H, *J* = 8.6 Hz), 7.33 (d, 1H, Ar-H, *J* = 8.9 Hz), 7.39–7.46 (m, 2 H, Ar-H), 7.84–7.93 (m, 3 H, Ar-H). ^13^C-NMR (75 MHz, DMSO-*d*_6_) δ_ppm_: 37.7, 55.4, 58.6, 114.5, 116.4, 117.2, 121.0, 124.1, 125.3, 127.4, 128.5, 128.9, 129.8, 130.6, 131.2, 138.3, 147.1, 158.3, 160.0. Anal. Calcd. for C_21_H_16_N_2_O_2_ (328.37): C, 76.81; H, 4.91; N, 8.53. Found: C, 76.87; H, 4.82; N, 8.61.

### Ethyl N-(2-cyano-1-(4-methoxyphenyl)-1H-benzo[f]chromen-3-yl)formimidate 2

A solution of enaminonitrile **1** (3.28 g, 10 mmol) and triethylorthoformate (20 mL, 50 mmol) was heated at reflux for 8 h. The formed solid was filtered off, washed with petroleum ether 60–80 °C, dried and then crystallized from ethanol/dioxane to give **2** as pale yellow crystals; yield 80%; mp 283–285 °C (lit. 284–286 °C); IR (KBr, ν, cm^− 1^): 3080 (CH, aromatic), 2973, 2936 (CH, aliphatic), 2213 (C ≡ N), 1656 (C = N). ^1^H NMR (300 MHz, DMSO-*d*_6_) δ_ppm_:1.20 (Syn), 1.31 (Anti) (2t, 3 H, CH_2_*CH*_*3*_, *J* = 7.2 Hz), 3.68 (s, 3 H, OCH_3_), 4.13 (Syn), 4.38 (Anti) 4.34 (q, 2 H, CH_3_*CH*_*2*_, *J* = 7.2 Hz), 5.51 (s, 1H, C_4_H-pyran), 6.84 (d, 2 H, Ar-H, *J* = 8.4 Hz), 7.18 (d, 2 H, Ar-H, *J* = 8.4 Hz), 7.40–7.47 (m, 4 H, Ar-H), 7.82–7.97 (m, 4 H, Ar-H), 8.18 (Syn), 8.70 (Anti) (2s, 1H, N = CH). ^13^C-NMR (75 MHz, DMSO-*d*_6_) δ_ppm_: 13.9, 18.9, 55, 56, 63.9, 81.4, 94.3, 114.0, 117.1, 118.0, 123.8, 125,1, 127.1, 128,7, 129.4, 129.80, 130.0, 131.20, 135.99 ,146.9, 156,6, 158,2, 161.8, 162.1. Anal. Calcd. for C_24_H_20_N_2_O_3_ (384.15): C, 74.98; H, 5.24; N, 7.29. Found: C, 74.91; H, 5.31; N, 7.35.

### (E)-N-(benzo[d]thiazol-2-yl)-N’-(2-cyano-1-(4-methoxyphenyl)-1H-benzo[f]chromen-3-yl)formimidamide 3

A mixture of formimidate derivative **2** (3.84 g, 10 mmol) and 2 aminobenzothiazole (10 mmol) in dry dioxan (20 mL) was refluxed for 12 h. After cooling, the formed solid was filtered off, dried, and then recrystallized from benzene/ethanol to give **3** as white crystals; yield 67%; mp 234–239 °C; IR (KBr, *ν*, cm^− 1^): 3178 (NH), 3043 (CH, aromatic), 2952 (CH, aliphatic), 2209 (C ≡ N), 1664 (C = N). ^1^H NMR (400 MHz, DMSO-*d*_6_) δ_ppm_: 3.68 (s, 3 H, OCH_3_), 5.55 (s, 1H, C_4_H-pyran), 6.86 (d, 2 H, Ar-H, *J* = 8.5 Hz), 7.21 (d, 2 H, Ar-H, *J* = 8.5 Hz), 7.44–7.50 (m, 5 H, Ar-H), 7.87 (d, 1H, Ar-H, *J* = 7.6 Hz), 7.93–7.99 (m, 4 H, Ar-H), 8.82 (s, 1H, N = CH), 11.12 (brs, 1H, NH, exchangeable with D_2_O). ^13^C-NMR (100 MHz, DMSO-*d*_6_) δ_ppm_: 38.6, 55.4, 114.7, 117.4, 124.20, 125.8, 127.7, 129.0, 130.2, 130.3, 131.8, 136.1, 146.9, 158.8. MS m/z (%): 488 (M.^+^, 20), 438 (30), 371 (18), 252 (16), 223 (53), 177 (64), 159 (35), 110 (63), 76 (100). Anal. Calcd. for C_29_H_20_N_4_O_2_S (488.57): C, 71.29; H, 4.13; N, 11,47; S, 6.56. Found: C, 71.21; H, 4.19; N, 11,41; S, 6.62.

### (E)-N’-(2-cyano-1-(4-methoxyphenyl)-1H-benzo[f]chromen-3-yl)-N-(1,5-dimethyl-3-oxo-2-phenyl-2,3-dihydro-1H-pyrazol-4-yl)formimidamide 4

 A mixture of formimidate derivative **2** (3.84 g, 10 mmol) and 4-aminoantipyrine (10 mmol) in Ethanol (20 mL) was refluxed for 10 h. After cooling, the formed solid was filtered off, dried, and then recrystallized from petroleum ether 60–80 °C/ benzene mixture to give **4** as white crystals; yield 75%; mp 284–286 °C; IR (KBr, *ν*, cm^− 1^): 3326 (NH, ), 3060 (CH, aromatic), 2932 (CH, aliphatic), 2194 (C ≡ N), 1659 (C = O). ^1^H NMR (300 MHz, DMSO-*d*_6_) δ_ppm_: 1.86 (s, 3 H, CH_3_), 3.08 (s, 3 H, CH_3_), 3.66 (s, 3 H, OCH_3_), 6.29 (s, 1H, C_4_H-pyran), 6.76–6.79 (m, 2 H, Ar-H), 7.31–7.55 (m, 10 H, Ar-H), 7.93–8.01 (m, 2 H, Ar-H + 1H, N = CH), 8.11 (d, 1H, Ar-H, *J* = 7.6 Hz), 8.56 (brs, 1H, NH, exchangeable with D_2_O). ^13^C-NMR (75 MHz, DMSO-*d*_6_) δ_ppm_: 10.3, 33.1, 35.9, 37.4, 55.0, 99.0, 108.5, 113.9, 114.1, 116.7, 116.9, 117.7, 117.8, 120.7, 123.2, 123.7, 124.9, 126.4, 127.1, 128.1, 128,4,128.5, 128.7, 129.2, 129.4, 130.2, 130. 9, 135.1, 135.8, 138.0, 146.8, 147.8, 153.5, 156.0, 157.9, 159.6, 160.7, 161.9, 162.5. MS m/z (%): 541 (M.^+^, 22), 532 (14), 518 (11), 442 (23), 422 (33), 349 (21), 259 (22), 152 (17), 116 (43), 97 (38), 71 (47), 57 (100). Anal. Calcd. for C_33_H_27_N_5_O_3_ (541.61): C, 73.18; H, 5.03; N, 12.93. Found: C, 73.24; H, 5.09; N, 12.87.

### (E)-N’-(2-cyano-1-(4-methoxyphenyl)-1 H-benzo[f]chromen-3-yl)-N-(5-nitropyridin-2-yl) formimidamide 5

A mixture of formimidate derivative **2** (3.84 g, 10 mmol) and 2-amino-5-nitropyridine (10 mmol) in dry dioxan (20 mL) was refluxed for 15 h. After cooling, the formed solid was filtered off, dried, and then recrystallized from petroleum ether 60–80 °C/ benzene to give 5 as white crystals; yield 55%; mp 176–178 °C; IR (KBr, *ν*, cm^− 1^): 3366 (NH), 2992, 2972 (CH, aliphatic), 2213 (C ≡ N), 1620 (C = N). ^1^H NMR (400 MHz, DMSO-*d*_6_) δ_ppm_: for anti-isomer [3.57 (s, 3 H, OCH_3_), 5.50 (s, 1H, C_4_H-pyran), 6.50 (d, 1H, ArH, *J* = 9.6 Hz), 6.85 (d, 3 H, ArH), 8.70 (s, 1H, N = CH)], for syn-isomer [3.67 (s, 3 H, OCH_3_), 6.75 (d, 1H, ArH, *J* = 8.8 Hz), 7.37 (d, 3 H, ArH), 8.85 (s, 1H, N = CH)], 7.41–7.57 (m, 8 H, Ar-H), 7.82–7.97 (m, 6 H, Ar-H), 8.86 (s, 1H, Ar-H), 10.65 (brs, 1H, NH, exchangeable with D_2_O). ^13^C-NMR (100 MHz, DMSO-*d*_6_) δ_ppm_: 14.3, 55.4, 64.3, 81.9, 107.6, 114.6, 117.5, 118.4, 124.2, 125.5, 127.5, 128.8, 128.9, 129.1, 130 (3), 131.6, 133.0, 134.8, 136.4, 139.4, 147.5, 156.2, 157.0, 158.7, 162.1, 163.7. MS m/z (%): 477 (M.^+^, 57), 466 (100), 431 (44), 371 (38), 335 (28), 318 (36), 299 (56), 263 (53), 202 (29), 174 (80), 134 (51), 76 (44). Anal. Calcd. for C_27_H_19_N_5_O_4_ (477,47): C, 67.92; H, 4.01; N, 14.67. Found: C, 67.86; H, 4.09; N, 14.72.

### 2-(11-imino-12-(4-methoxyphenyl)-11 H-benzo[5,6]chromeno[2,3-d]pyrimidin-10(12 H)-yl)ethan-1-ol 6

A mixture of formimidate derivative 2 (3.84 g, 10 mmol) and ethanolamine (0.66 mL, 10 mmol) in dry pyridine (20 mL) was refluxed for 3 h. After cooling, the formed solid was filtered off, dried, and then recrystallized from benzene/ethanol to give **6** as white crystals; yield 60%; mp 188–190 °C; ; IR (KBr, ν, cm-1): 3396 (NH), 2999 (CH, aromatic), 2931 (CH, aliphatic), 1633 (C = N). 1 H NMR (400 MHz, DMSO-*d*6) δppm: 3.56 (t, 4 H, CH_2_-CH_2_), 3.61 (s, 3 H, OCH_3_), 4.78 (s, 1 H, OH, exchangeable with D_2_O), 6.06 (s, 1 H, C_4_H-pyran), 6.76 (d, 2 H, Ar-H, *J* = 8.7 Hz), 7.36–7.45 (m, 2 H, Ar-H + 1 H, C = NH, exchangeable with D_2_O), 7.47–7.61 (m, 3 H, Ar-H), 7.93 (d, 2 H, Ar-H, *J* = 8.7 Hz), 8.21 (s, 1 H, N = CH), 8.24 (d, 1 H, Ar-H, *J* = 8.5 Hz).^13^C-NMR (100 MHz, DMSO-*d*6) δppm: 33.3, 55.3, 59.9, 98.3, 114.3, 118.0, 118.3, 123,7, 125.3, 127.4, 129.0, 130.9, 131.2, 136.2, 148.1, 156.4, 158.4, 161.0, 161.8. MS m/z (%): 399 (M.^+^, 23), 371 (92), 361 (100), 344 (98), 331 (50), 263 (47), 236 (57), 229 (53), 203 (57), 198 (28), 173 (29), 156 (40), 118 (42), 96 (72), 87 (49), 78 (37). Anal. Calcd. for C_24_H_21_N_3_O_3_ (399.45): C, 72.17; H, 5.30; N, 10.52. Found: C, 72.21; H, 5.25; N, 10.47.

### 1-(11-imino-12-(4-methoxyphenyl)-11 H-benzo[5,6]chromeno[2,3-d]pyrimidin-10(12 H)-yl) urea 7

A mixture of formimidate derivative **2** (3.84 g, 10 mmol) and semicarbazide hydrochloride (10 mmol) in Ethanol (20 mL) containing a catalytic amount of fused sodium acetate (0.2 g) was refluxed for 11 h. After cooling, the formed solid was filtered off, dried, and then recrystallized from ethanol to give **7** as white crystals; yield 65%; mp 214–216 °C; IR (KBr, *ν*, cm^− 1^): 3368, 3182 (NH, NH_2_), 2930, 2834 (CH, aliphatic), 1674 (C = O), 1630 (C = N). ^1^H NMR (400 MHz, DMSO-*d*_6_) δ_ppm_: 3.61 (s, 3 H, OCH_3_), 5.88 (brs, 2 H, NH_2_, exchangeable with D_2_O), 6.19 (s, 1H, C_4_H-pyran), 6.77 (d, 2 H, Ar-H, *J* = 8.6 Hz), 7.36–7.94 (m, 4 H, Ar-H + 1H, NH, exchangeable with D_2_O), 7.96–8.22 (m, 3 H, Ar-H), 8.23 (d, 1H, Ar-H, *J* = 8.5 Hz), 8.28 (s, 1H, N = CH), 9.22 (brs, H, NH, exchangeable with D_2_O). ^13^C-NMR (100 MHz, DMSO-*d*_6_) δ_ppm_: 33.2, 55.4, 98.6, 114.3, 118.0, 118.4, 123.6, 125.4, 127.5, 129.1, 129.7, 130.8, 131.3, 136.0, 148.1, 156.4, 158.3, 162.1. MS m/z (%): 413 (M.^+^, 40), 351 (64), 339 (96), 336 (100), 311 (53), 264 (37), 239 (65), 205 (67), 143 (42), 132 (47), 93 (24), 70 (24). Anal. Calcd. for C_23_H_19_N_5_O_3_ (413.44): C, 66.82; H, 4.63; N, 16.94. Found: C, 66.89; H, 4.71; N, 16.55.

### 2-(14-(4-methoxyphenyl)-14 H-benzo[5,6]chromeno[3,2-e] [1,2,4] triazolo[1,5-c]pyrimidin-2-yl)acetonitrile 8

A mixture of formimidate derivative **2** (3.84 g, 10 mmol) and cyanoacetohydrazide (10 mmol) in dry dioxan (20 mL) was refluxed for 10 h. After cooling, the formed solid was filtered off, dried, and then recrystallized from benzene to give **8** as reddish yellow crystals; yield 54%; mp 208–210 °C; IR (KBr, *ν*, cm^− 1^): 3060 (CH, aromatic), 2959, 2926 (CH, aliphatic), 2255 (C ≡ N), 1634 (C = N). ^1^H NMR (300 MHz, DMSO-d_6_) δ_ppm_: 3.61 (s, 3 H, OCH_3_), 4.49 (s, 2 H, *CH*_*2*_CN), 6.25 (s, 1H, C_4_H-pyran), 6.75 (d, 2 H, Ar-H, *J* = 8.4 Hz), 7.34–7.62 (m, 5 H, Ar-H), 7.95–8.11 (m, 3 H, Ar-H), 9.63 (s, 1H, N = CH_pyrimidine_). ^13^C-NMR (75 MHz, DMSO-*d*_6_) δ_ppm_: 18.1, 36.1, 55.0, 102.5, 114.0, 115.3, 116.6, 117.5, 123.6, 125.2, 127.4, 128.4, 128.7, 129.3,129.9,130.3, 131.2, 135.2, 140.2, 147.8, 153.0, 153.4, 158,1, 160.9. MS m/z (%): 419 (M.^+^, 25), 379 (31), 351 (51), 342 (100), 309 (47), 236 (45), 159 (17), 107 (45), 91 (26). Anal. Calcd. for C_25_H_17_N_5_O_2_ (419.14): C, 71.59; H, 4.09; N, 16.70. Found: C, 71.48; H, 4.19; N, 16.61.

### (E)-3-(4-chlorophenyl)-2-(14-(4-methoxyphenyl)-14 H-benzo[5,6]chromeno[3,2-e] [1,2,4] triazolo[1,5-c]pyrimidin-2-yl)acrylonitrile 9

A mixture of compound **8** (2.1 g, 5 mmol) and *p*-chlorobenzaldehyde (0.7 mL, 5 mmol) in dry dioxan (30 mL) was heated at reflux for 6 h. The produced solid while reflux was filtered off, dried, and then recrystallized from ethanol to yield **9** as red crystals; yield 72%; mp > 300 °C; IR (KBr, *ν*, cm^− 1^): 3073, 3032 (CH aromatic), 2996 (CH aliphatic), 2220 (C ≡ N), 1635 (C = N). ^1^H NMR (400 MHz, DMSO-*d*_6_) δ_ppm_: 3.61 (s, 3 H, OCH_3_), 6.30 (s, 1H, C_4_H-pyran), 6.78–8.15 (m, 14 H, Ar-H), 9.69 (s, 1H, N = *CH*_Pyrimidine_), ^13^C-NMR (100 MHz, DMSO-*d*_6_) δ_ppm_: 36.6, 55.4, 103.1, 114.4, 115.4, 116.1, 117.9, 123.9, 125.6, 127.9, 128.7, 129.1, 129.7, 130.4, 130.7, 131.7, 132.2, 135.4, 136.0, 137.3, 140.9, 147.9, 148.4, 153.3, 153.9, 158.5, 162.5. MS m/z (%): 542 (M.^+^, 26), 491 (90), 448 (56), 413 (39), 354 (74), 324 (74), 267 (70), 179 (57), 139 (100), 91 (95), 79 (18), 77 (41). Anal. Calcd. for C_32_H_20_ClN_5_O_2_ (542.00): C, 70.91; H, 3.72; Cl, 6.54; N, 12.92. Found: C, 70.98; H, 3.65; Cl, 6.61; N, 12.86.

### 14-(4-methoxyphenyl)-14 H-benzo[5,6]chromeno[3,2-e] [1,2,4] triazolo[1,5-c]pyrimidin-2-amine 10

A mixture of formimidate derivative **2** (3.84 g, 10 mmol) and thiosemicarbazide (0.91 g, 10 mmol) in pyridine (20 mL) was refluxed for 10 h. After cooling, the formed material was filtered off, dried, and then recrystallized from benzene to give **10** as brown crystals; yield 60%; mp 265–267 °C; IR (KBr, *ν*, cm^− 1^): br 3485, 3315 (NH_2_), 3080, 3061 (CH, aromatic), 2955, 2929 (CH, aliphatic), 1651 (C = N). ^1^H NMR (400 MHz, DMSO-*d*_6_) δ_ppm_: 3.61 (s, 3 H, OCH_3_), 6.0 (s, 1H, C_4_H-pyran), 6.77 (d, 2 H, Ar-H, *J* = 8.6 Hz), 7.17 (brs, 2 H, NH_2_, exchangeable with D_2_O), 7.36–7.56 (m, 5 H, Ar-H, *J* = 8.4 Hz), 7.93 (d, 2 H, Ar-H, *J* = 8.6 Hz), 8.11 (s, 1H, N = CH_Pyrimidine_), 8.22 (d, 1H, Ar-H, *J* = 8.5 Hz). ^13^C-NMR (100 MHz, DMSO-*d*_6_) δ_ppm_: 33.8, 55.3, 97.7, 114.2, 118.1, 118.5, 123.6, 125.3, 127.4, 128.7, 129.1, 129.6, 130.9, 131.2, 136.3, 148.2, 156.6, 158.3, 162.3, 162.9. MS m/z (%): 395 (M.^+^, 53), 387 (67), 371 (64), 325 (100), 290 (72), 283 (59), 179 (52), 150 (40), 128 (76), 85 (74), 78 (40). Anal. Calcd. for C_23_H_17_N_5_O_2_ (395.42): C, 69.86 ; H, 4.33; N, 17.71. Found. C, 69.94; H, 4.25; N, 17.89.

### Insect rearing

*Culex pipiens* colony was maintained under controlled conditions as a laboratory strain at the insectary of the Entomology Department, Faculty of Science, Ain Shams University. Mosquitoes were reared at 26–28 °C, 70–80% RH and a 14: 10 h (L/D)^81^. Adults were reared in (40 × 40 × 40 cm) insect cage and fed on 10% sucrose solution prepared in distilled water. To sustain egg production, females were fed on pigeon twice a week for half an hour. Egg rafts were collected from ovipositional jars and transferred to plastic pans containing one liter of distilled water and were fed fish food. Pupae were picked from larval rearing pans and were transferred to the adult rearing cage daily^82^.

### Larvicidal bioassay activity

Larvicidal efficacy of the synthesized compounds was tested against the third larval instar of *C. pipiens*. A stock solution of each synthesized compound was prepared in Dimethyl Sulfoxide (DMSO) while temephos (LARVIGUARD^®^) 50% EC, Shri Ram Agro Chemicals, India) (WHO recommended larvicide) was dissolved in water. This stock was used to prepare six dilutions of all tested compounds (400, 300, 200, 100, 50 and 25 ppm) through distilled water. A 100-mL plastic cup was filled with 60 mL of each concentration. Twenty-third larval instars were placed into each cup according to the WHO protocol^83^ with a few modifications. Three replicates of each treatment were established. Parallel three-control replicates were conducted by adding DMSO to distilled water without any other chemicals. Following a 48-hour exposure, the number of dead larvae was recorded^84^.

### Adulticidal bioassay activity

The effectiveness of synthesized compounds was evaluated against adult mosquitoes using the CDC bottle bioassays^85^ with some modifications. Six concentrations for each tested compound were conducted using DMSO as a solvent. Deltamethrin (deltamethrin 98%; Rudong Zhongyi Chemical Co., Ltd, Rudong, China) was used as a positive control (WHO-recommended) and dissolved in water. For each concentration of all tested compounds (25, 50, 100, 200, 300 and 400 ppm), three bottles were coated with residual film of the tested compounds, then left overnight for solvent evaporation. Ten mixed-sex adults of *C. pipiens* (aged 4–5 days) fed on sucrose solution (10%), were transferred to each bottle by aspirator then left for an hour as exposure time. The mosquito groups were transferred from the treatment bottles to corresponding cups containing pads immersed in sugar solution^7^. Control was made by coating the bottles with DMSO only and was replicated three times. Adult deaths were observed after 24 h of recovery.

### Cytotoxicity assay

The human lung fibroblast cell line (WI-38) was obtained from ATCC via biological products and vaccines (VACSERA) company, Cairo, Egypt. Using the MTT assay, the normal cell line was employed to investigate the synthesized benzochromene’s inhibitory effects on cell growth. In this experiment, mitochondrial succinate dehydrogenase in the living cells causes the yellow tetrazolium bromide (MTT) to change into a purple formazan derivative. These cells were incubated in RPMI-1640 medium with 10% of fetal bovine serum^86^. After incubation, the normal cells were treated with (100, 50, 25, 12.5, 6.25, 3.12 and 1.56 µM) of the most effective compounds 5 and 10 and incubated for 24 h, then 20 µL of MTT solution at (5 mg/mL) was added and incubated again for 4 h. Dimethyl sulfoxide (DMSO) (in the volume of 100 µL) is mixed with each well to dissolve the formed purple formazan. The colorimetric assay is measured at an absorbance of (570 nm) using a plate reader (EXL 800, USA). The relative cell viability was calculated as ((A570) of treated samples/(A570) of untreated sample) X 100^87^.

### In silico studies

#### Molecular docking

A molecular docking analysis has been performed to investigate the ligand-receptor interactions of the benzochromene derivatives with acetylcholinesterase (AChE) of *Anopheles gambiae*. Benzochromene derivatives were drawn by ChemOffice Ultra 2004 (https://chemoffice-ultra-2004software.informer.com). The AChE of *A. gambiae* acquired from the protein data bank (https://www.rcsb.org/ ), AChE (PDB code: 5YDJ). The protein binding site was detected, and active pocket was found by the Deepsite at PlayMolecule^®^ platform (https://www.playmolecule.com/deepsite/*)*^88^. The 3D structures of the benzochromene derivatives, reference insecticide (Temephos) and ligand were geometrically optimized and energy-minimized at MMFF94X force field (0.05 kcal /mol) using Avogadro 1.2.0 software^89^. The protein (AChE) was prepared and docked with benzochromene derivatives and reference insecticide (Temephos); in addition, redocking of ligand using AutoDock Vina package^90^. The five poses with the least docking energies of tested compounds were used to visualize their alignment and binding interaction in AChE pocket by PyMOL software (https://www.pymol.org/ )^91^. The confirmations were evaluated and ranked depending on the London ΔG energy scoring function^78^.

#### DFT and MEP

The synthesized compounds were subjected to Density Functional Theory (DFT) calculations utilizing the B3LYP functional and the 6-311 + + G(d, p) basis set with GAUSSIAN 09 W software. The accuracy of this method in assessing molecule stability and reactivity is well known^92^. GaussView 5.0 was used to visualize molecular structures^93^. Geometry optimizations were performed without imposing any symmetry constraints. Quantum chemical analyses included assessments of the molecular electrostatic potential (MEP), optimized structures, and molecular orbital (MO) energy levels.

### Statistical analysis

The mortality percentage of various concentrations was recorded, and toxicity data were estimated according to Finney probit analysis^94^. LC values with fiducial limits, slope with standard errors and chi-square values of the probit regression lines were calculated by the LDP line package (Ehab©, Egypt). The relative potency of the tested compounds was estimated according to Villeneuve et al.^95^.

## Supplementary Information

Below is the link to the electronic supplementary material.

Supplementary Material 1

## Data Availability

All data generated or analyzed during this study are included in this published article and its supplementary information files.
