# Supplementary material for: Synthesis, SAR, and in silico studies of new benzochromene derivatives as insecticidal agents against Culex pipiens L. larvae and adults
Source: Sci Rep. 2025 Dec 9;15:43485. doi: 10.1038/s41598-025-30027-z (PMC12695882; doi:10.1038/s41598-025-30027-z)
Supplement: Supplementary file 1 — Supplementary Material 1 [file 41598_2025_30027_MOESM1_ESM.docx]

**
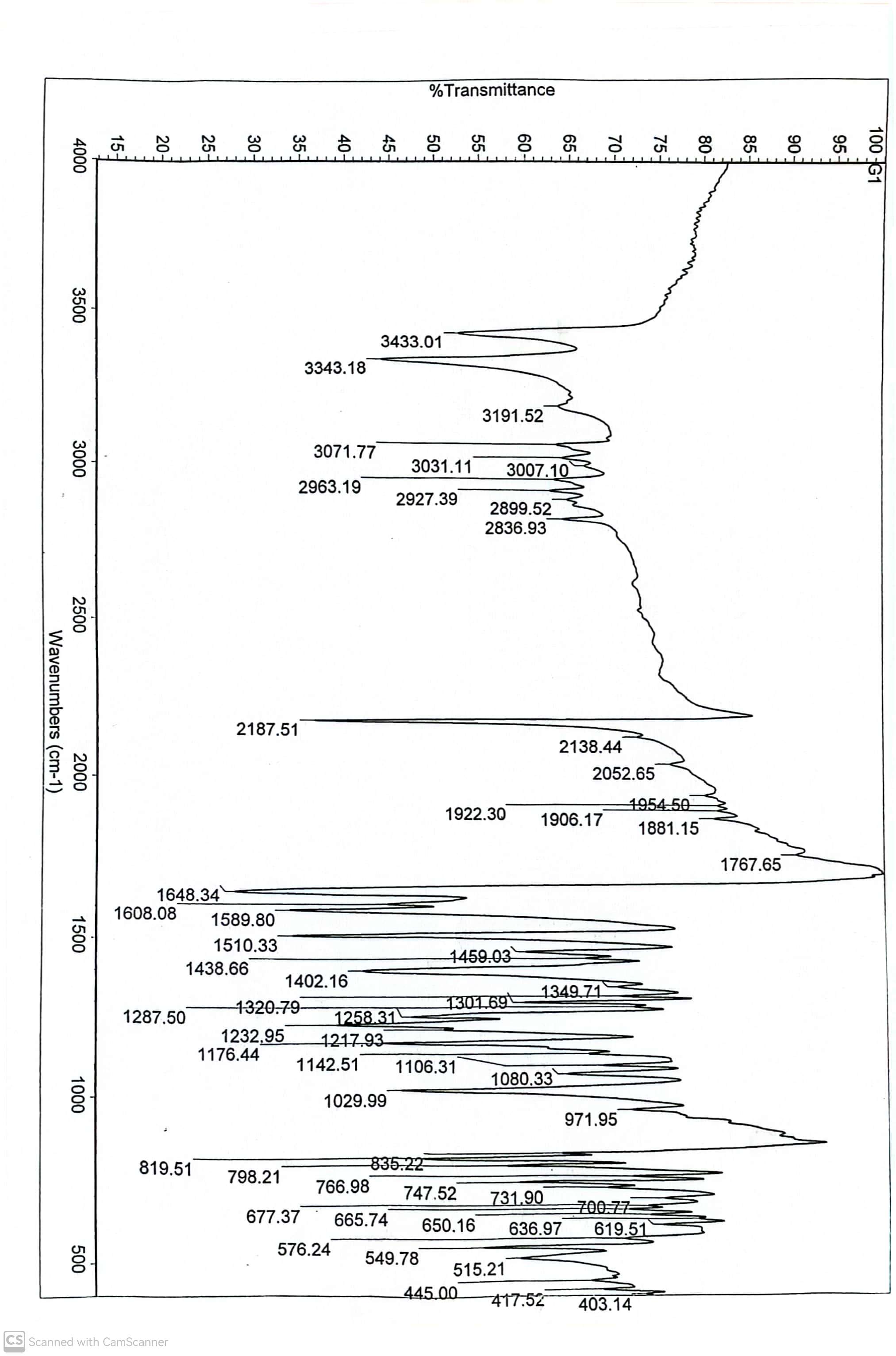
**

**Fig. S1: IR spectrum of compound 1**

**
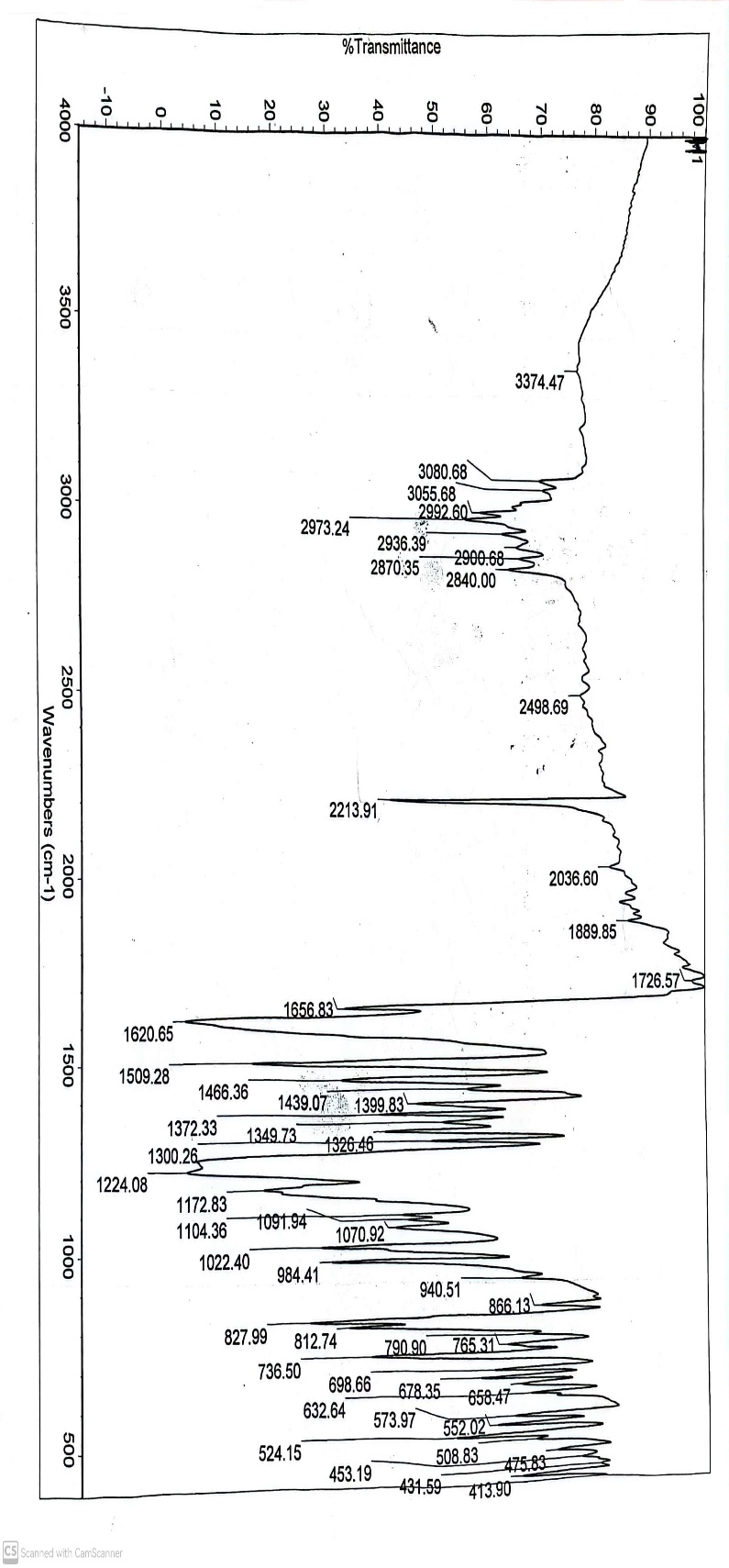
**

**Fig. S2: IR spectrum of compound 2**


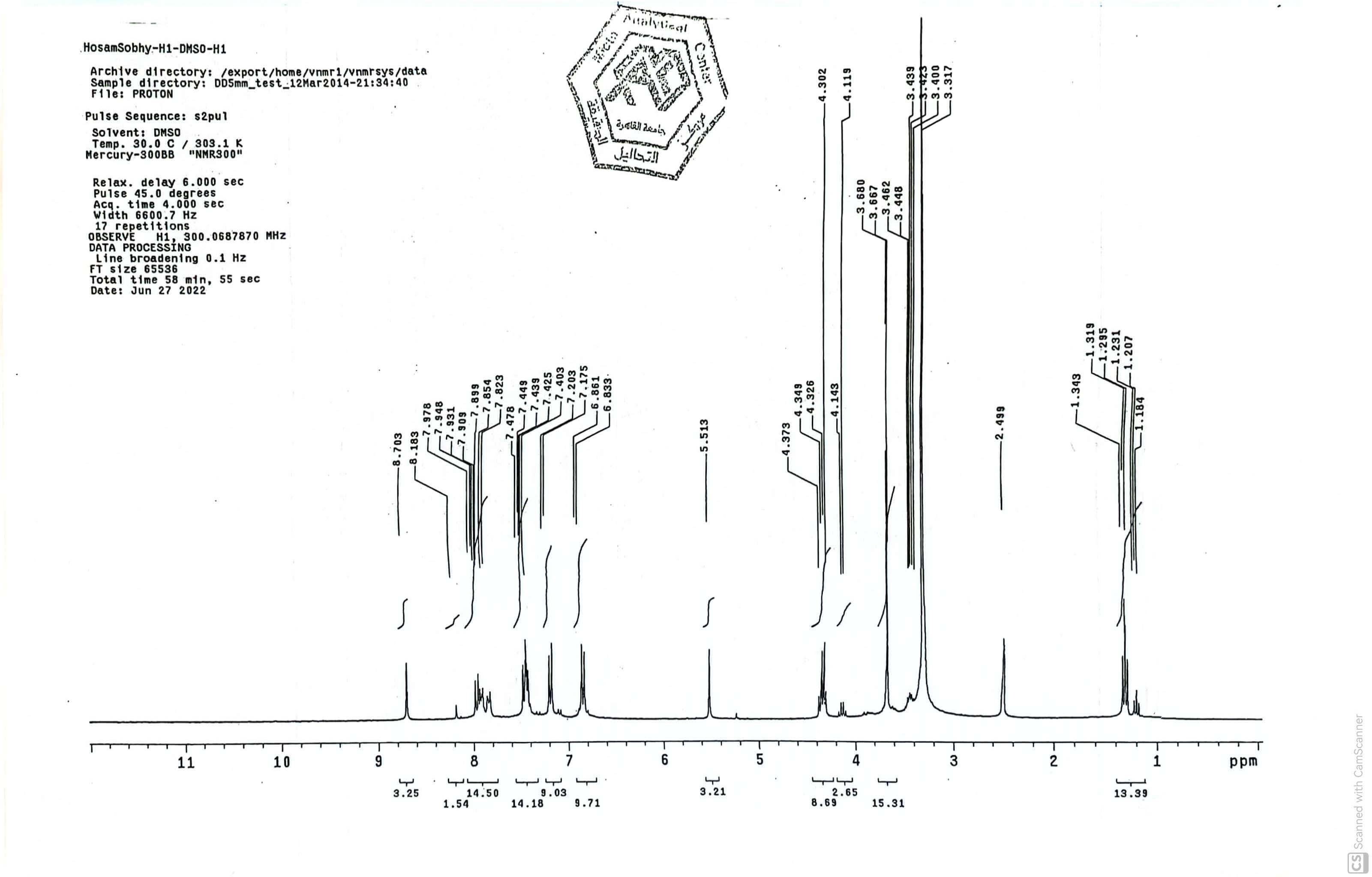


**Fig. S3: ^1^H-NMR spectrum (DMSO-d_6_) of compound 2**

**
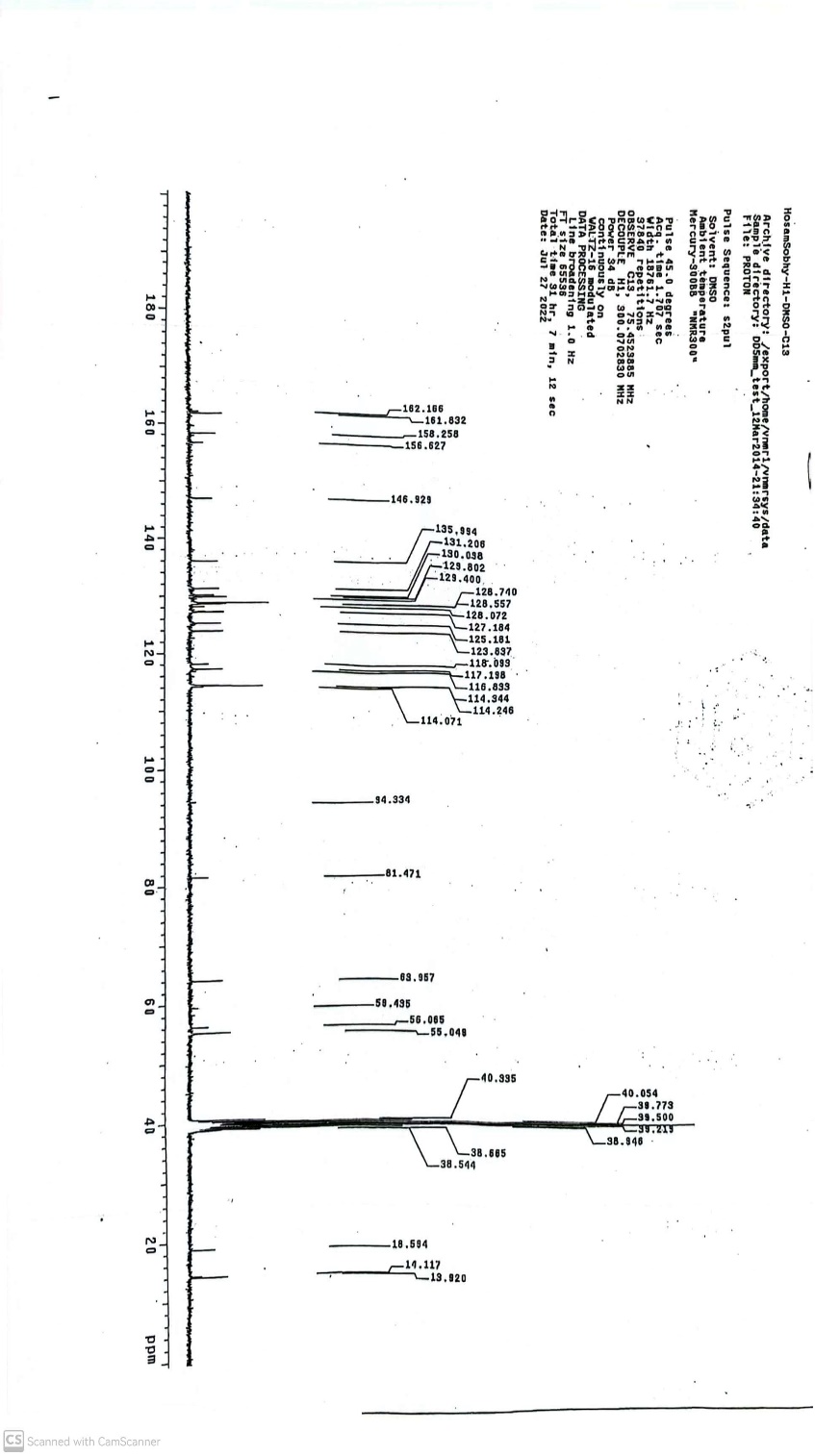
**

**Fig. S4: ^13^C-NMR spectrum (DMSO-*d*_6_) of compound 2**

**
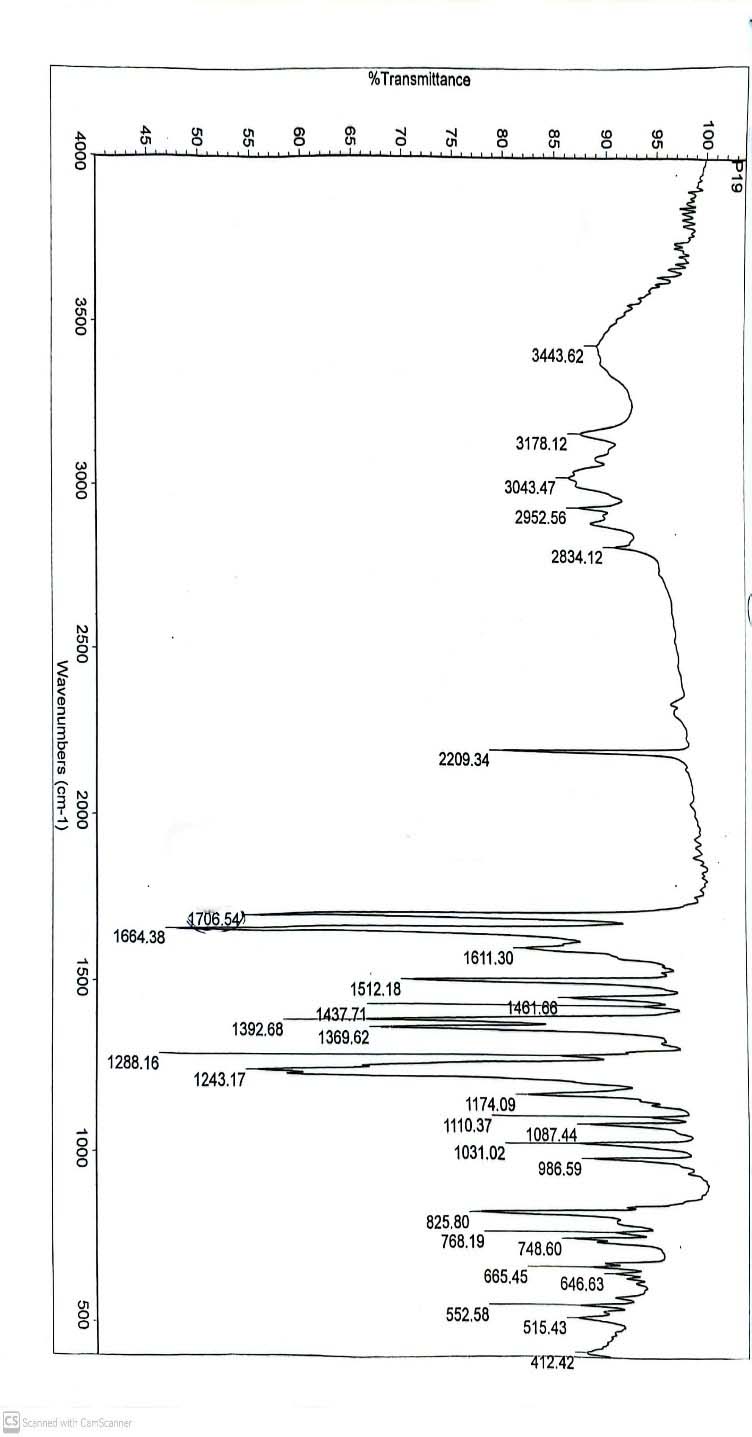
**

**Fig. S5: IR spectrum of compound 3**

**
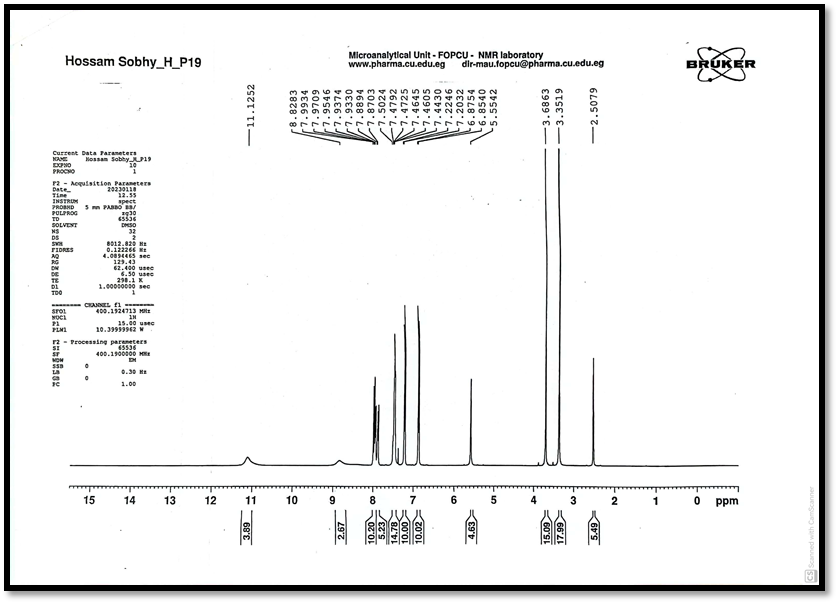
**

**Fig. S6: ^1^H-NMR spectrum (DMSO-d_6_) of compound 3**

**
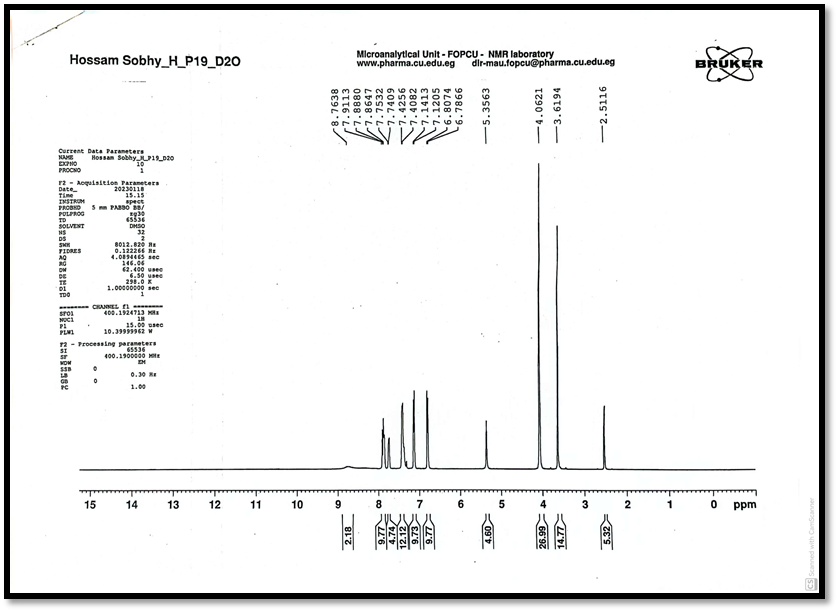
**

**Fig. S7: ^1^H-NMR spectrum (DMSO-*d*_6_+D_2_O) of compound 3**

**
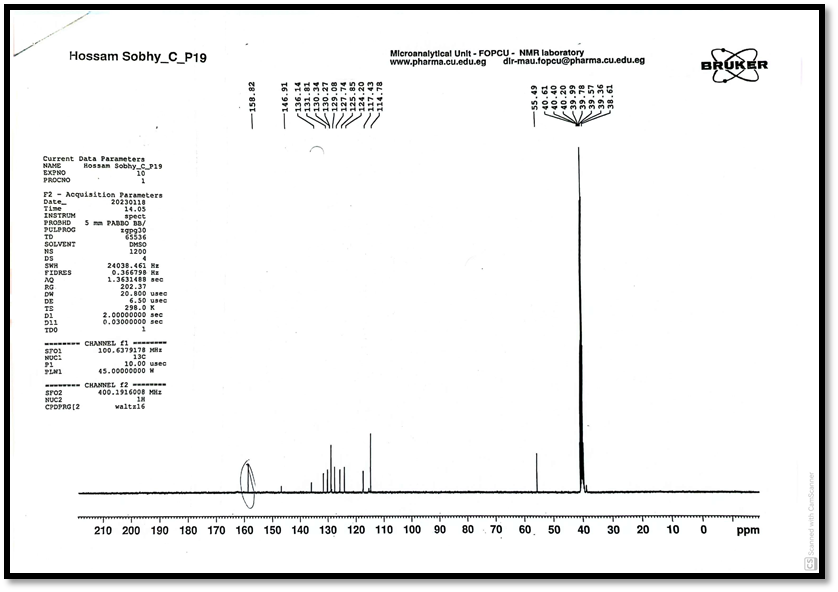
**

**Fig. S8: ^13^C-NMR spectrum (DMSO-d_6_) of compound 3**

**Fig. S9: Mass spectrum of compound 3**

**
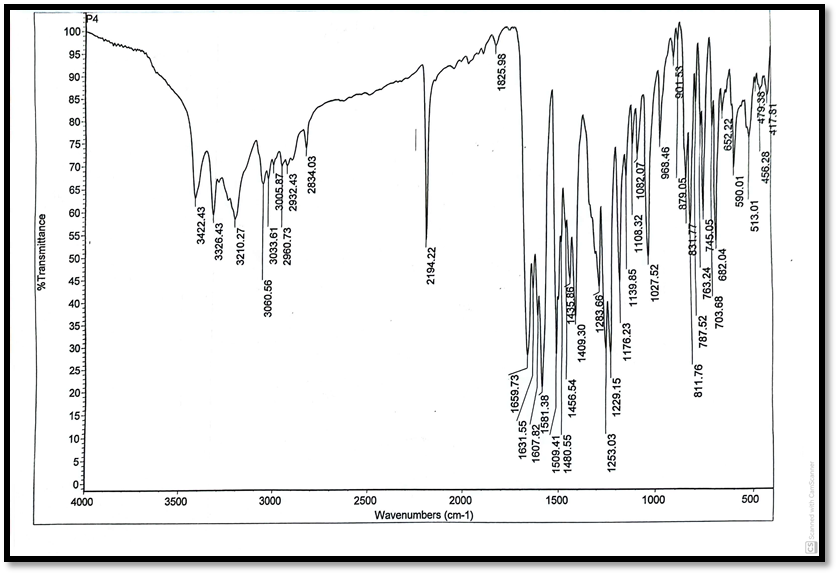
**

**Fig. S10: IR spectrum of compound 4**


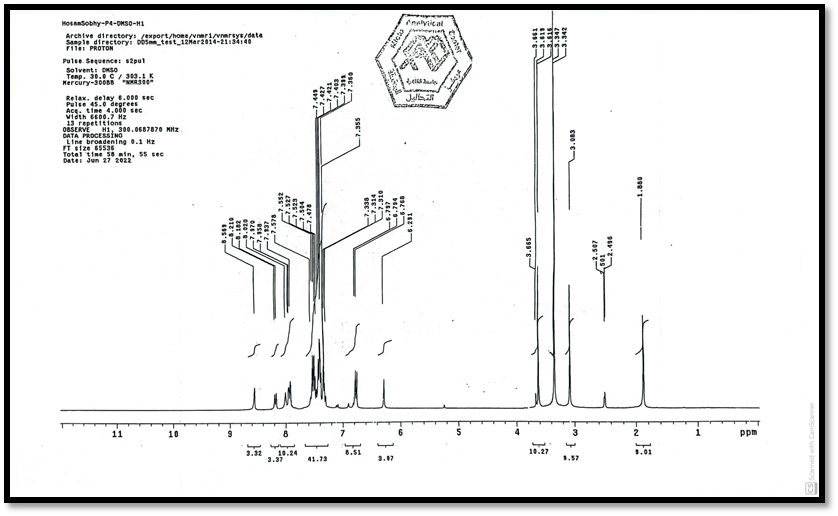


**Fig. S11: ^1^H-NMR spectrum (DMSO-d_6_) of compound 4**

**
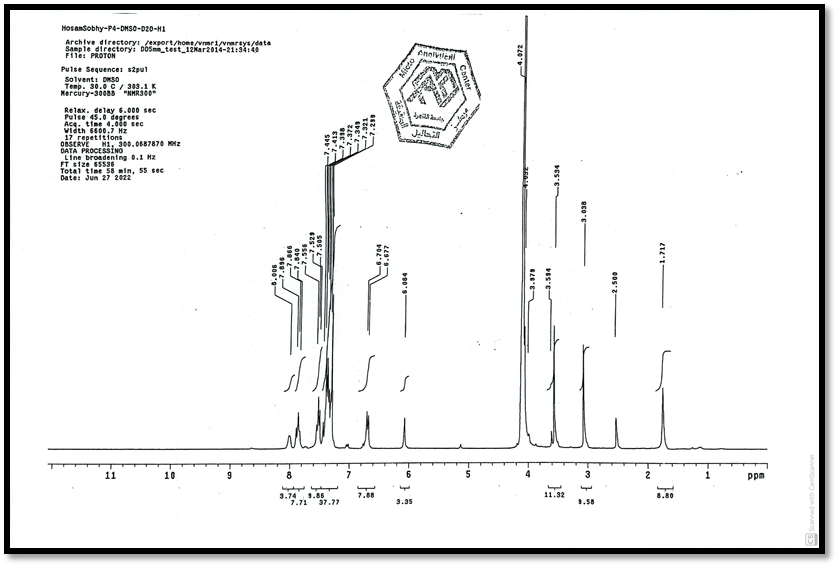
**

**Fig. S12: ^1^H-NMR spectrum (DMSO-*d*_6_ + D_2_O) of compound 4**

**
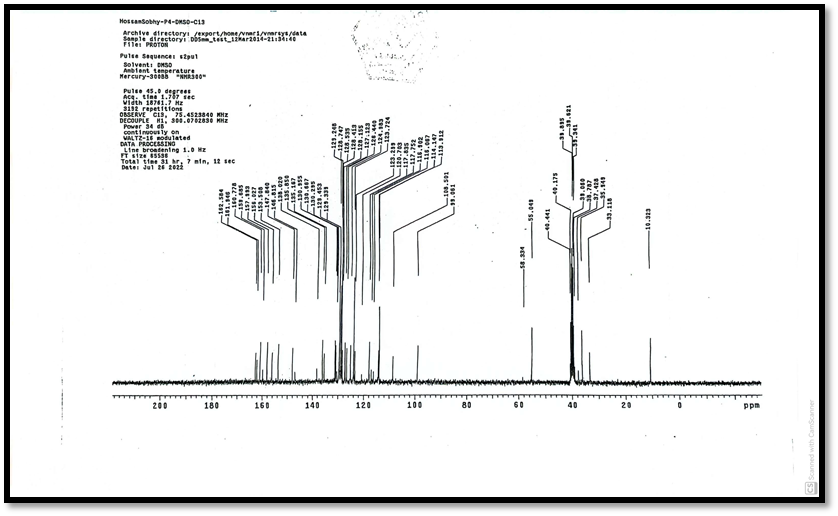
**

**Fig. S13: ^13^C-NMR spectrum (DMSO-d_6_) of compound 4**

**Fig. S14: Mass spectrum of compound 4**

**
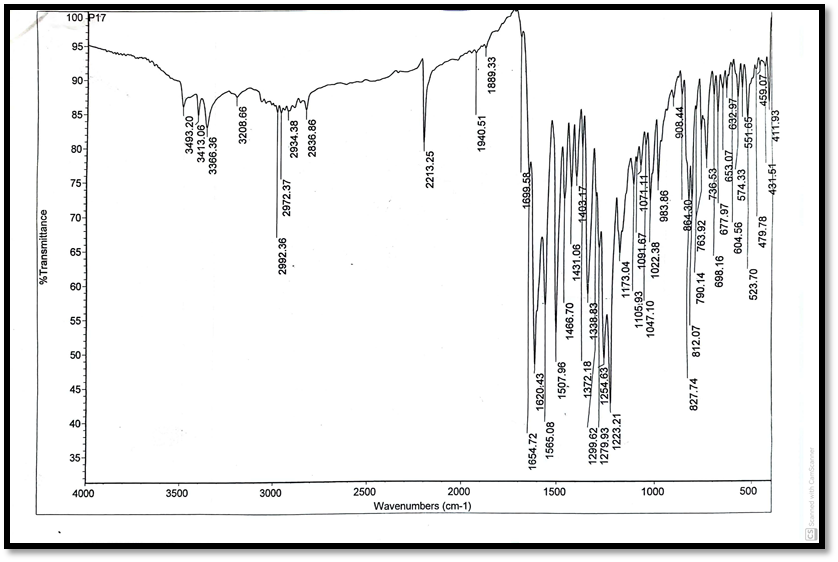
**

**Fig. S15: IR spectrum of compound 5**


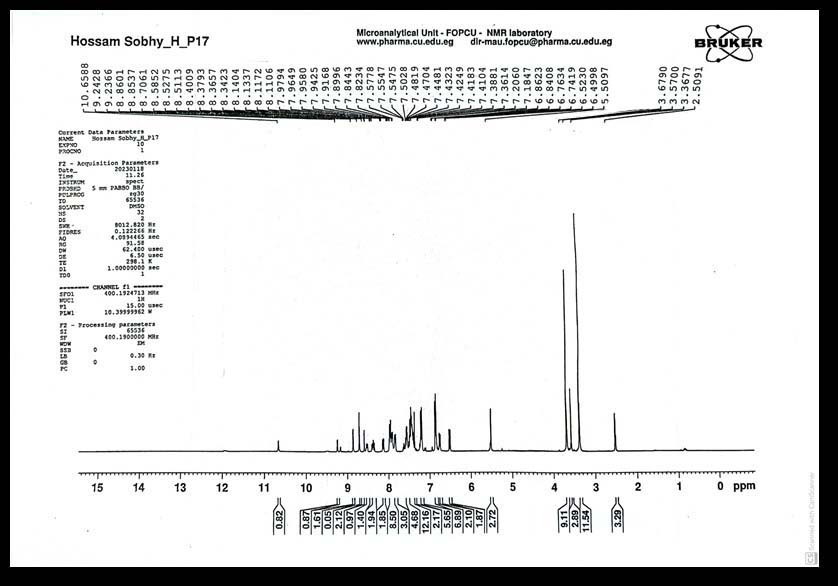


**Fig. S16: ^1^H-NMR spectrum (DMSO-d_6_) of compound 5**

**
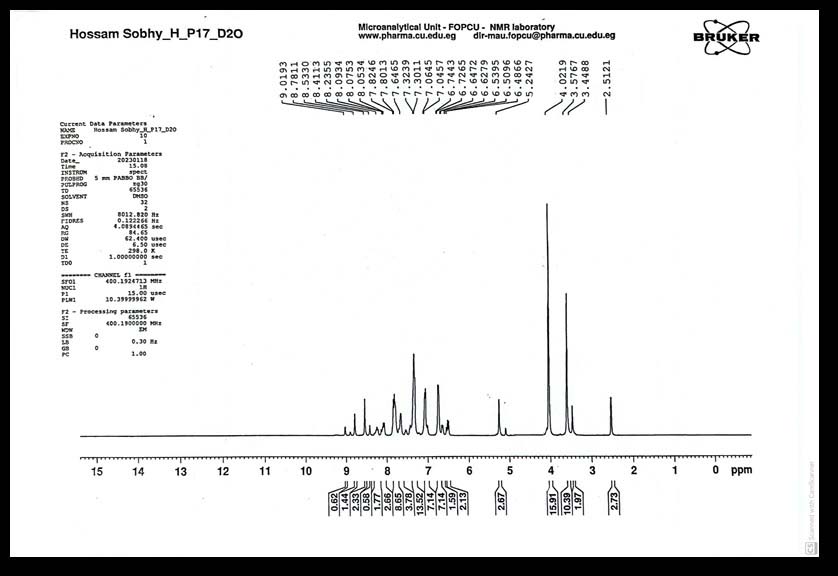
**

**Fig. S17: ^1^H-NMR spectrum (DMSO-d_6_ + D_2_O) of compound 5**

**
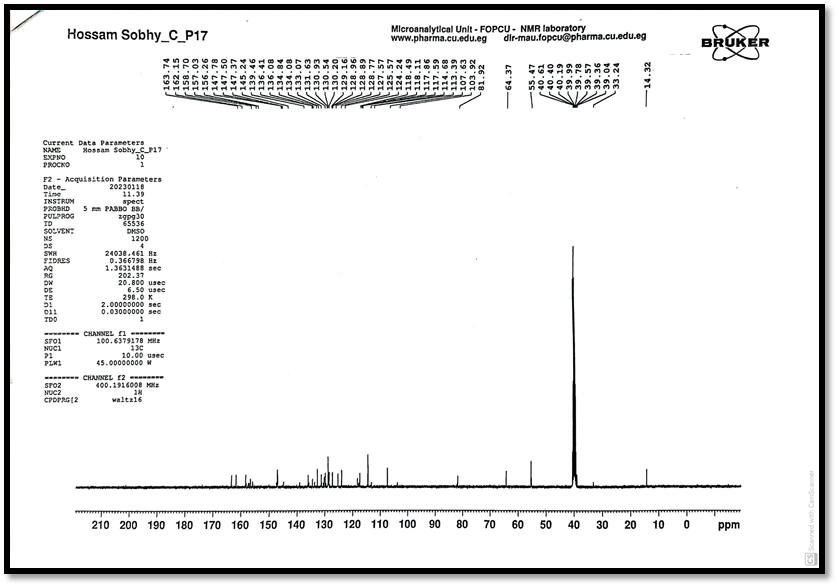
**

**Fig. S18: ^13^C-NMR spectrum (DMSO-d_6_) of compound 5**

**Fig. S19: Mass spectrum of compound 5**

**
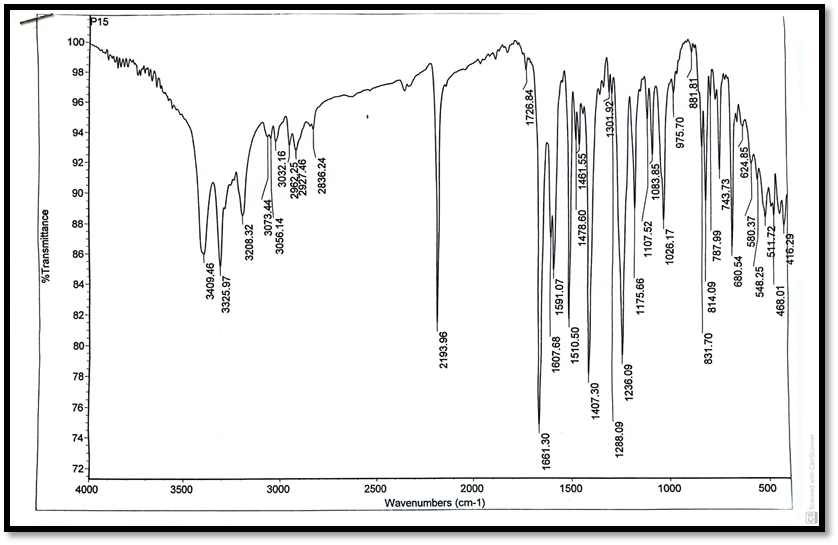
**

**Fig. S20: IR spectrum of compound 1**

**
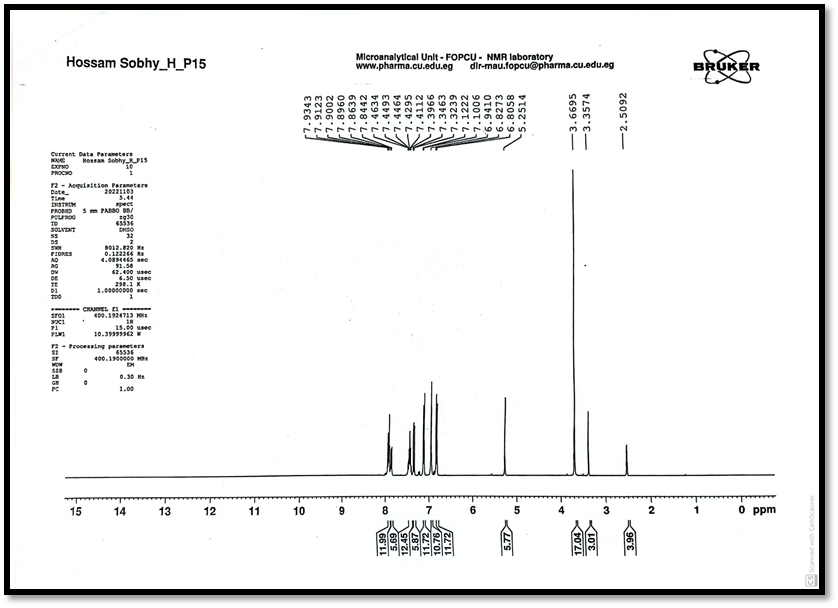
**

**Fig. S21: ^1^H-NMR spectrum (DMSO-d_6_) of compound 1**

**
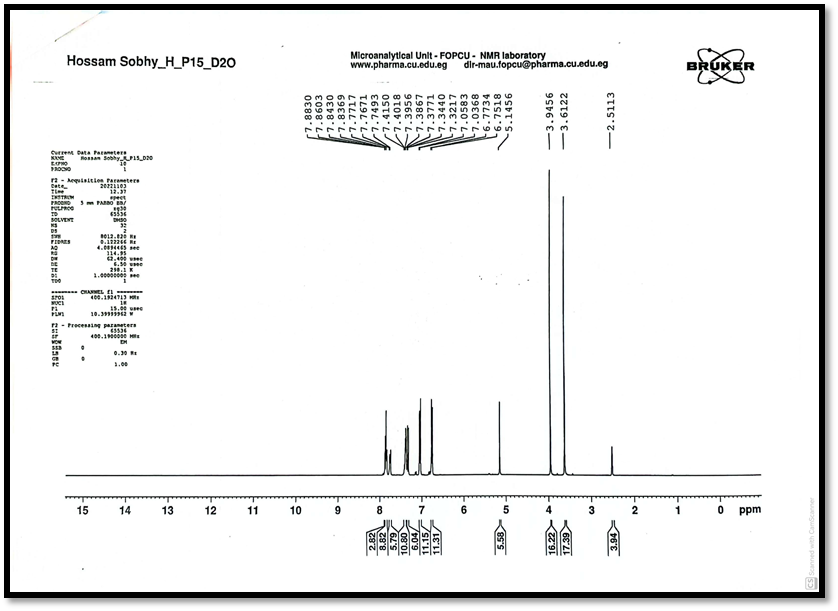
**

**Fig. S22: ^1^H-NMR spectrum (DMSO-d_6_ + D_2_O) of compound 1**

**
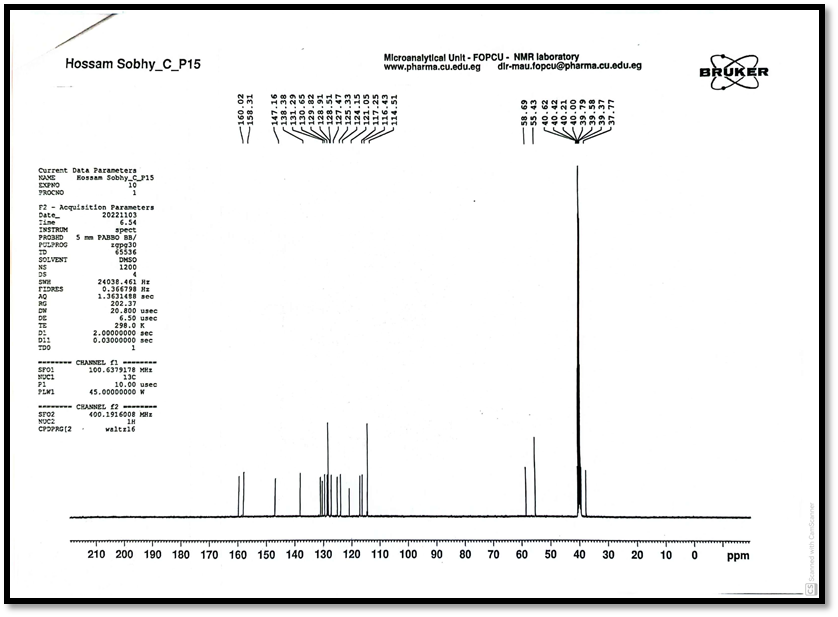
**

**Fig. 23: ^13^C-NMR spectrum (DMSO-d_6_) of compound 1**

**
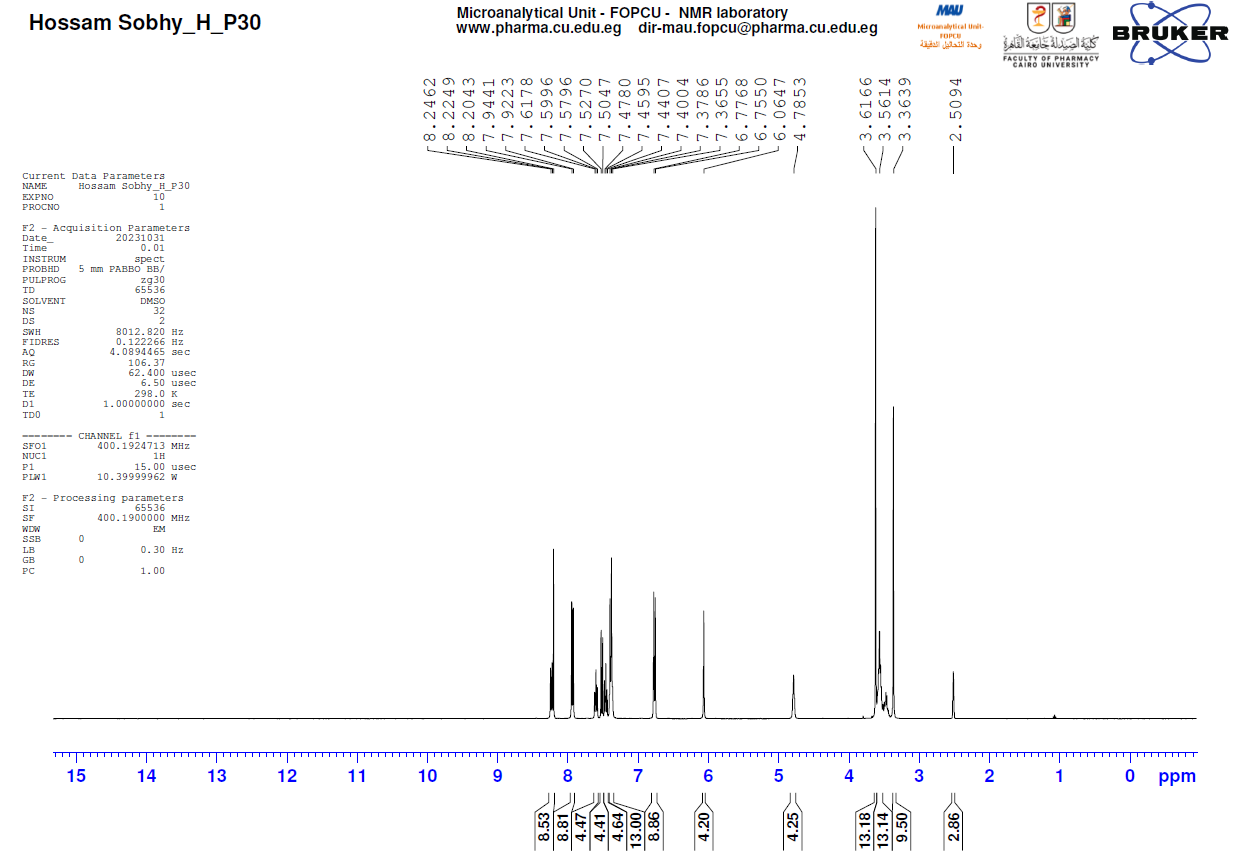
**

**Fig. S24: ^1^H-NMR spectrum (DMSO-d_6_) of compound 6**

**
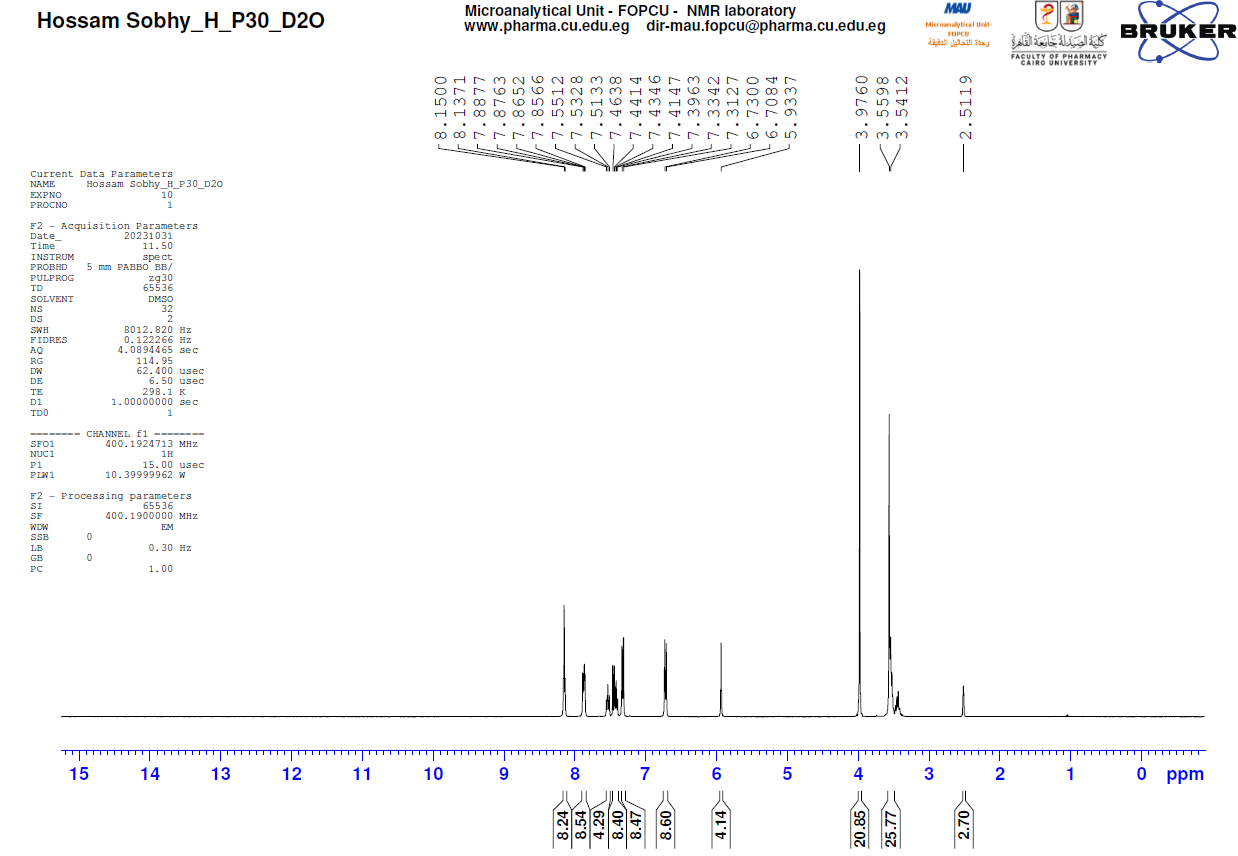
**

**Fig. S25: ^1^H-NMR spectrum (DMSO-d_6_ + D_2_O) of compound 6**

**
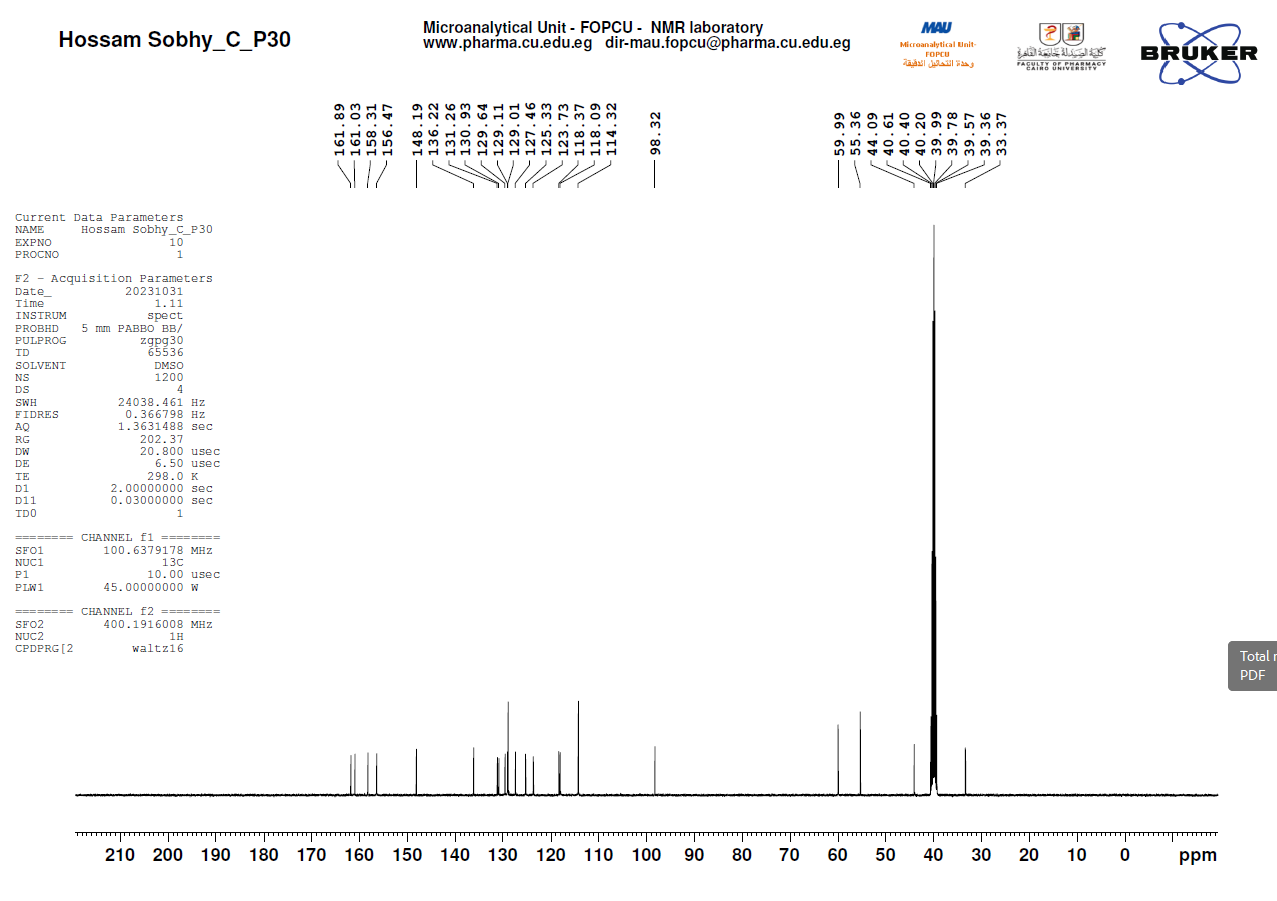
**

**Fig. S26: ^13^C-NMR spectrum (DMSO-d_6_) of compound 6**

**Fig. S27: Mass spectrum of compound 6**

**
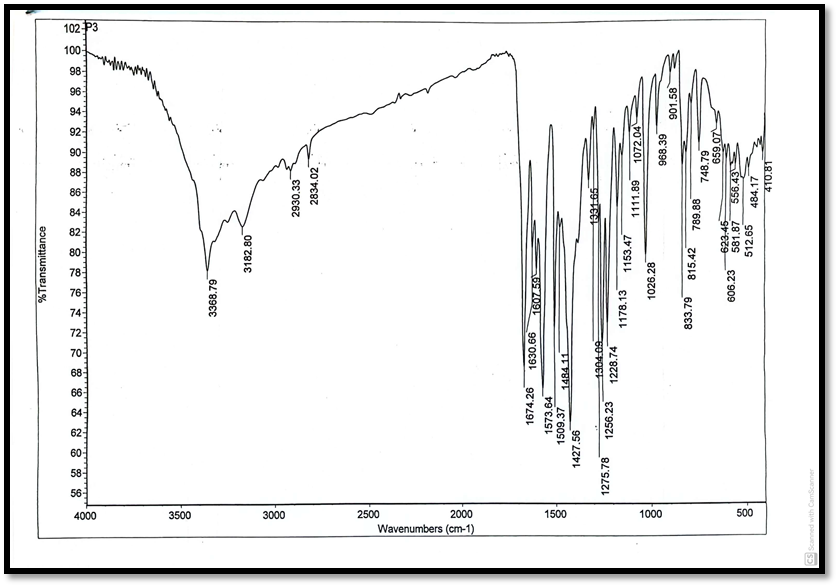
**

**Fig. S28: IR spectrum of compound 7**

**
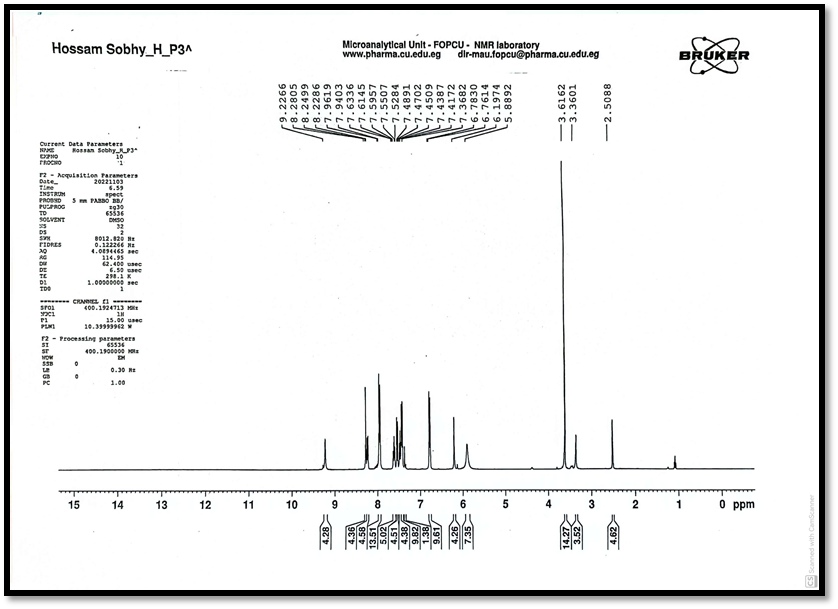
**

**Fig. S29: ^1^H-NMR spectrum (DMSO-d_6_) of compound 7**

**
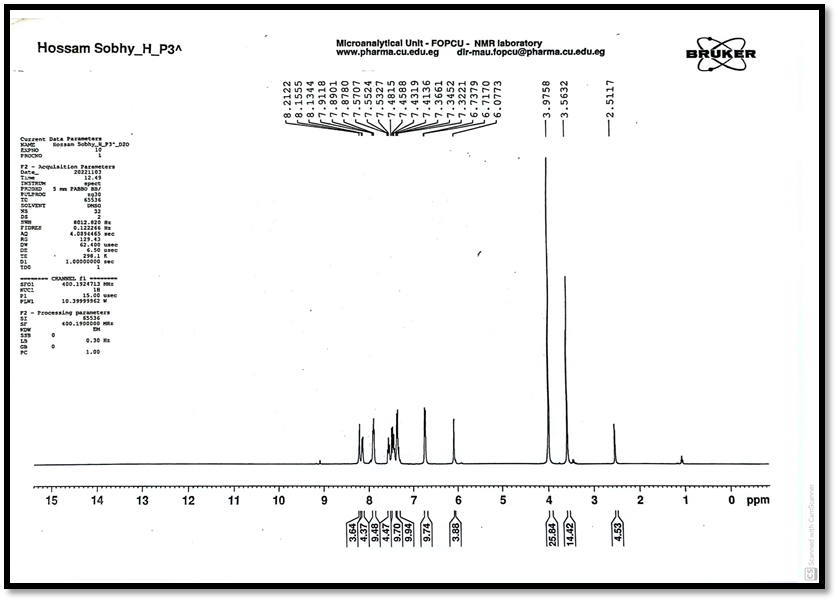
**

**Fig. S30: ^1^H-NMR spectrum (DMSO-d_6_ + D_2_O) of compound 7**

**
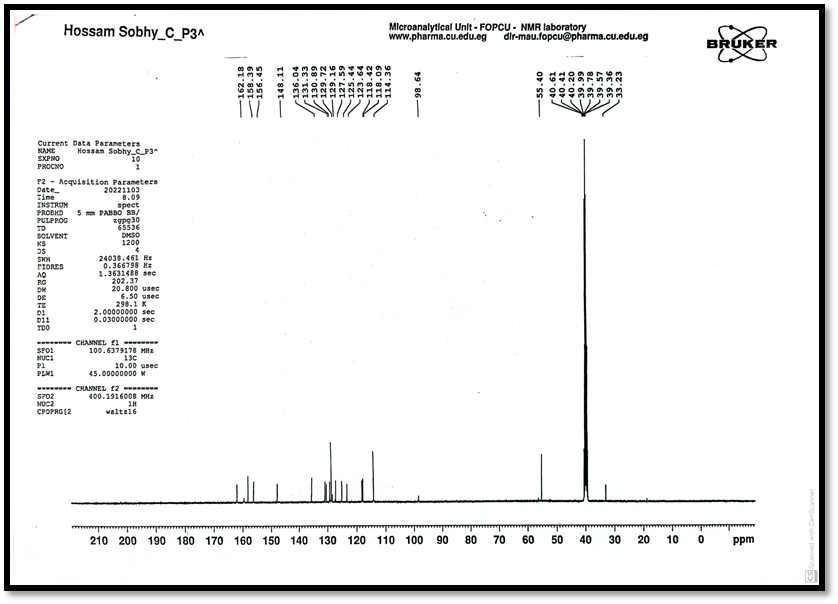
**

**Fig. S31: ^13^C-NMR spectrum (DMSO-d_6_) of compound 7**

**Fig. S32: Mass spectrum of compound 7**

**
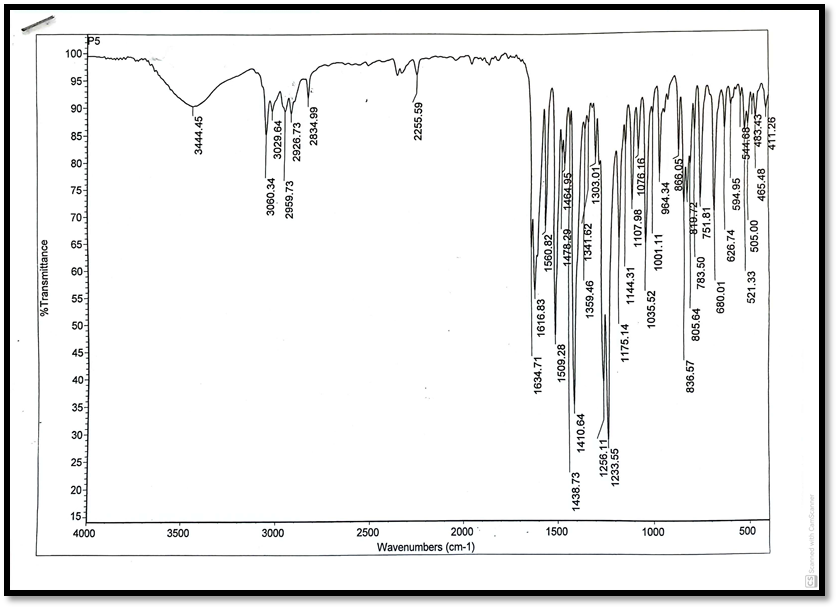
**

**Fig. S33: IR spectrum of compound 8**

**
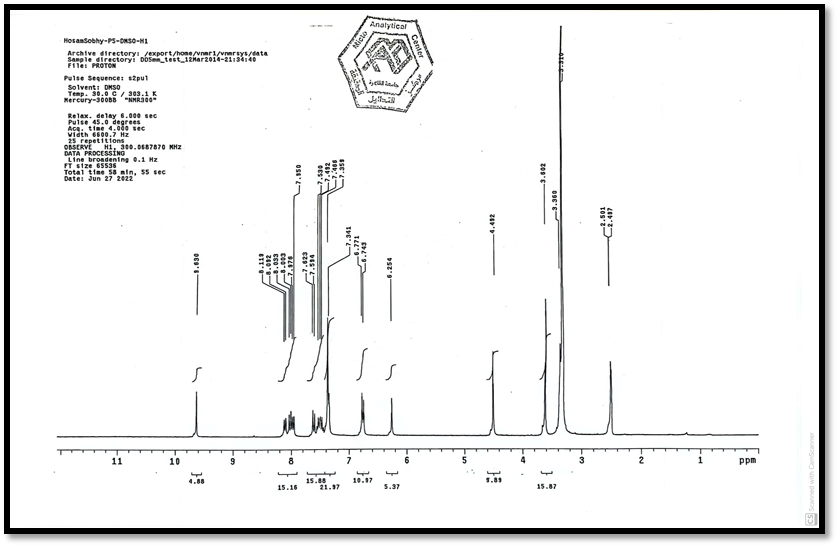
**

**Fig. S34: ^1^H-NMR spectrum (DMSO-d_6_) of compound 8**

**
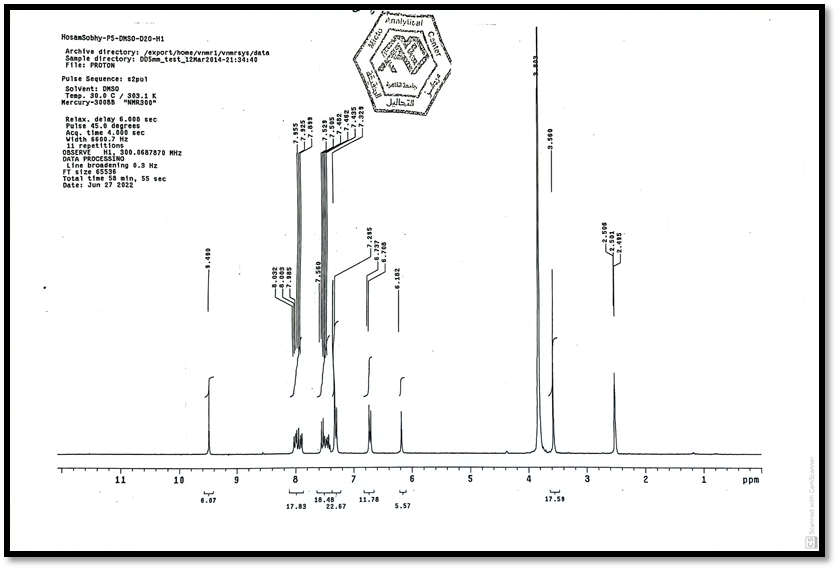
**

**Fig. S35: ^1^H-NMR spectrum (DMSO-d_6_ + D_2_O) of compound 8**

**
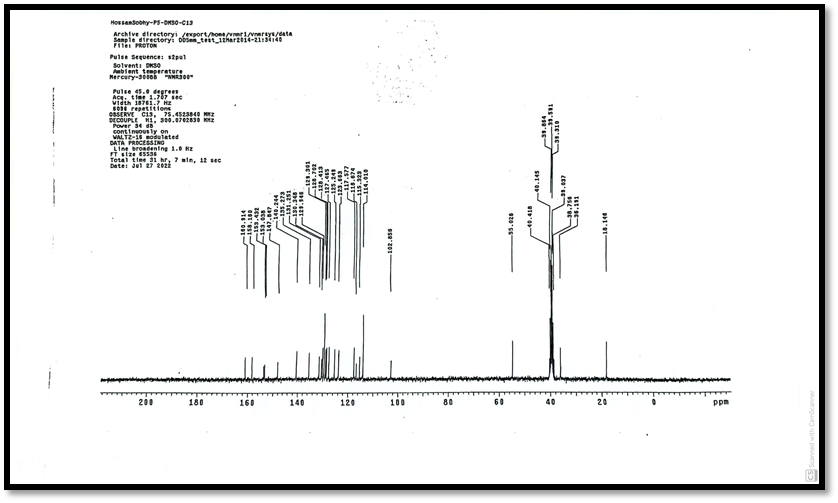
**

**Fig. S36: ^13^C-NMR spectrum (DMSO-d_6_) of compound 8**

**Fig. S37: Mass spectrum of compound 8**

**
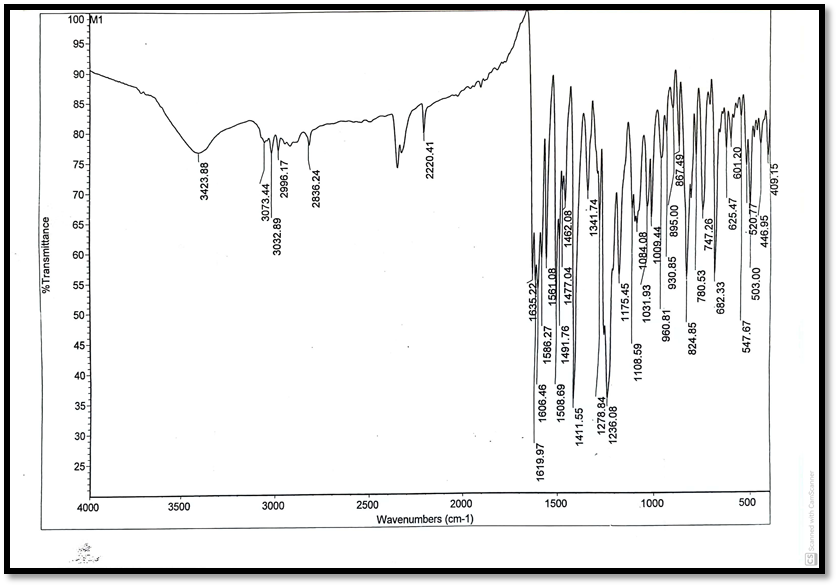
**

**Fig. S38: IR spectrum of compound 9**

**
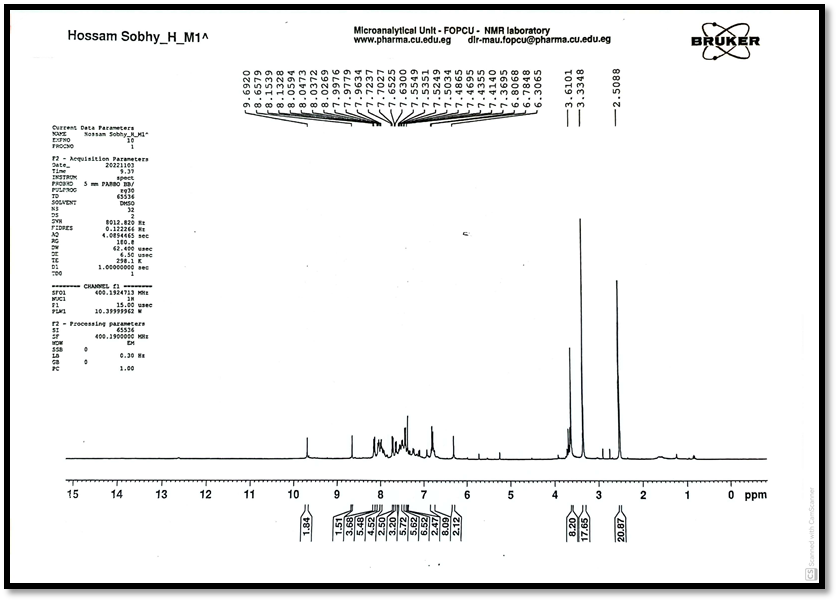
**

**Fig. S39: ^1^H-NMR spectrum (DMSO-d_6_) of compound 9**

**
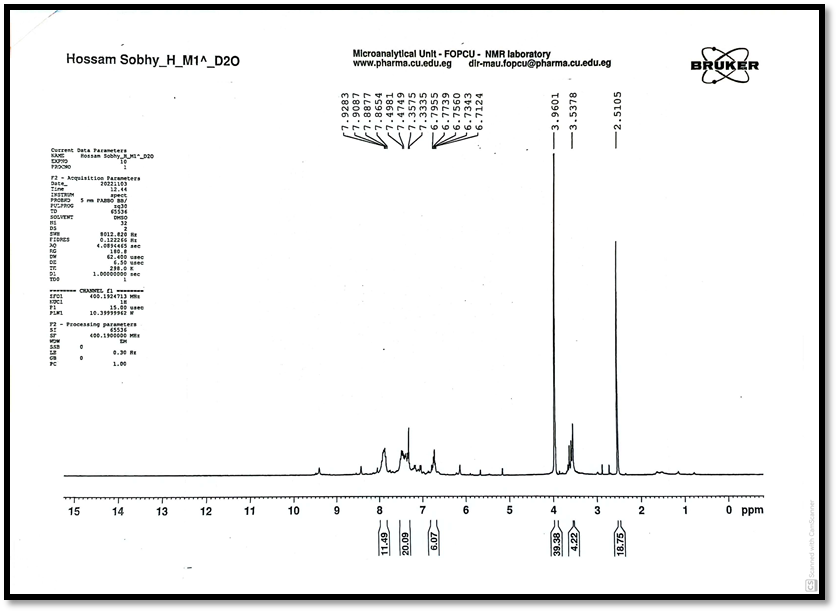
**

**Fig. S40: ^1^H-NMR spectrum (DMSO-d_6_+D_2_O) of compound 9**

**
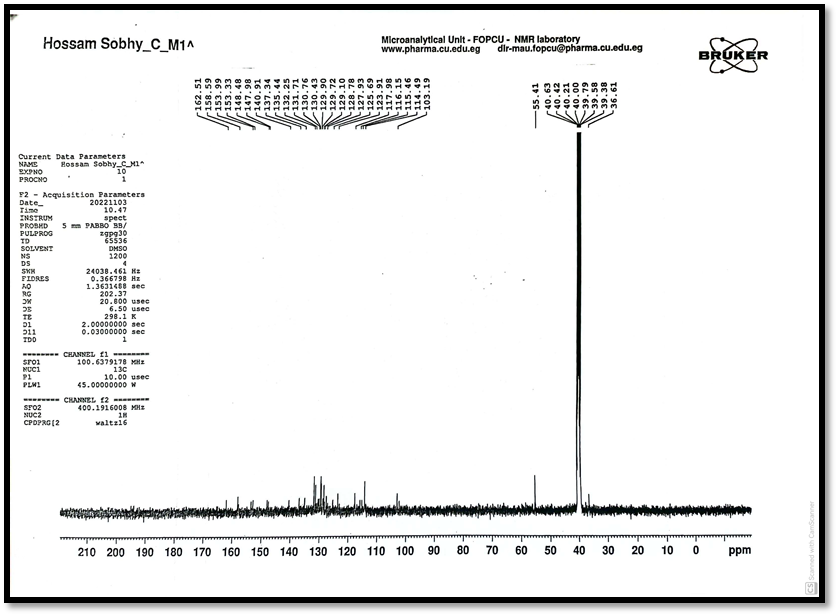
**

**Fig. S41: ^13^C-NMR spectrum (DMSO-d_6_) of compound 9**

**Fig. S42: Mass spectrum of compound 9**

**
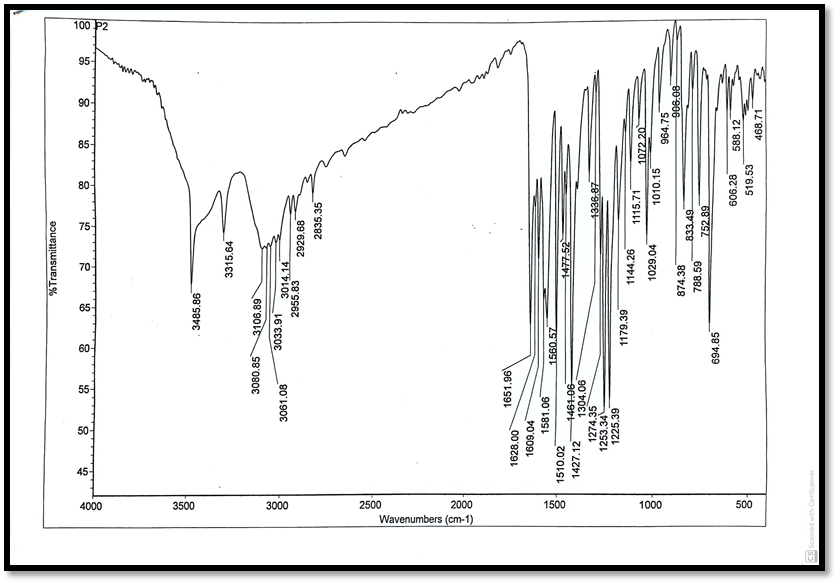
**

**Fig. S43: IR spectrum of compound 10**

**
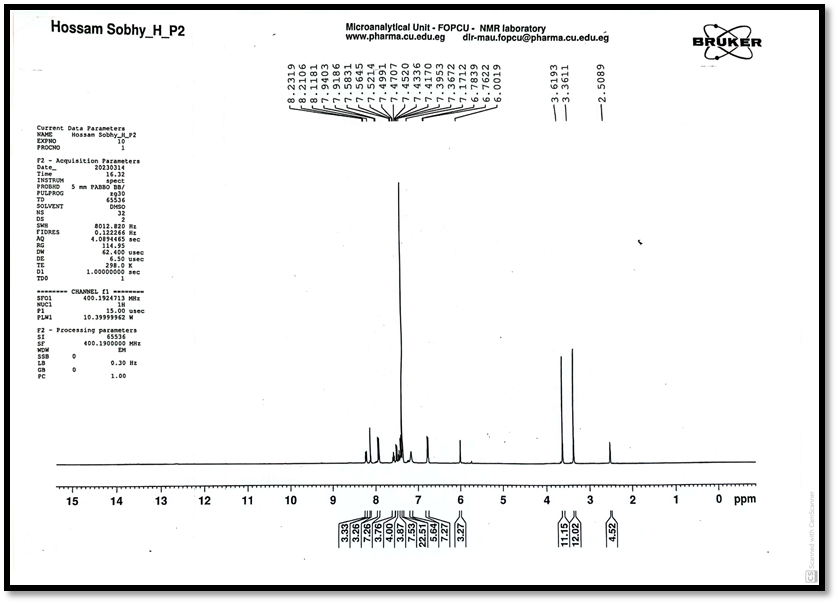
**

**Fig. S44: ^1^H-NMR spectrum (DMSO-d_6_) of compound 10**

**
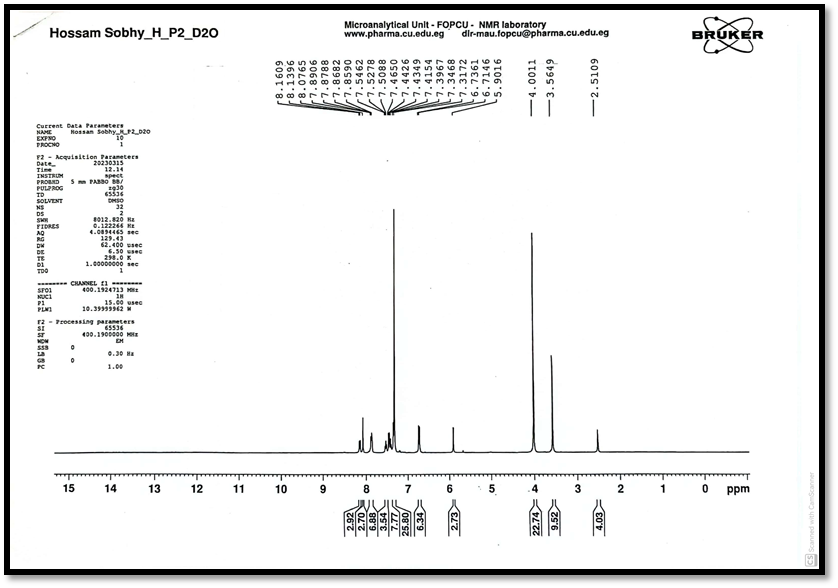
**

**Fig. S45: ^1^H-NMR spectrum (DMSO-d_6_ + D_2_O) of compound 10**

**
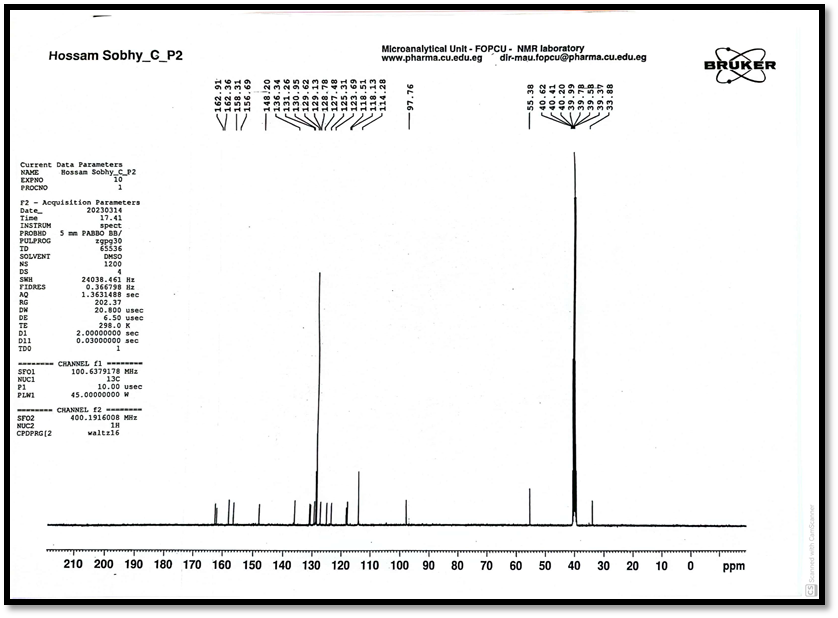
**

**Fig. S46: ^13^C-NMR spectrum (DMSO-d_6_) of compound 10**

**Fig. S47: Mass spectrum of compound 10**

| **3**  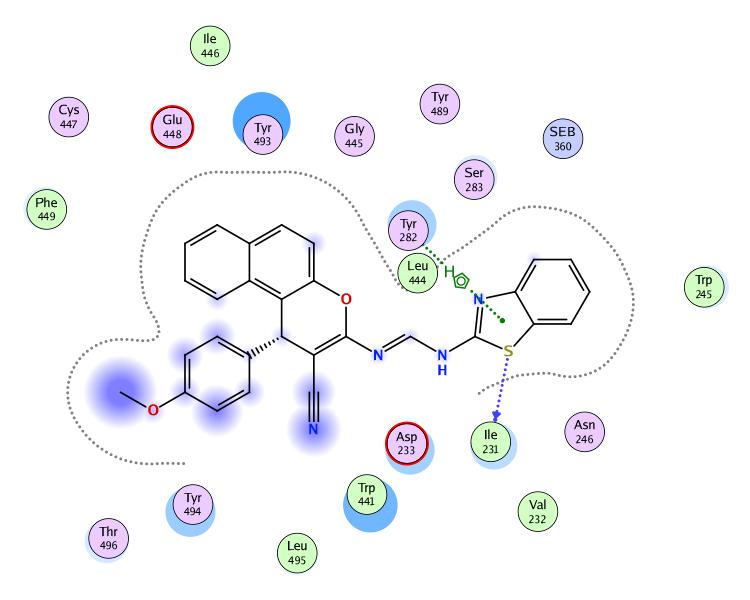 | 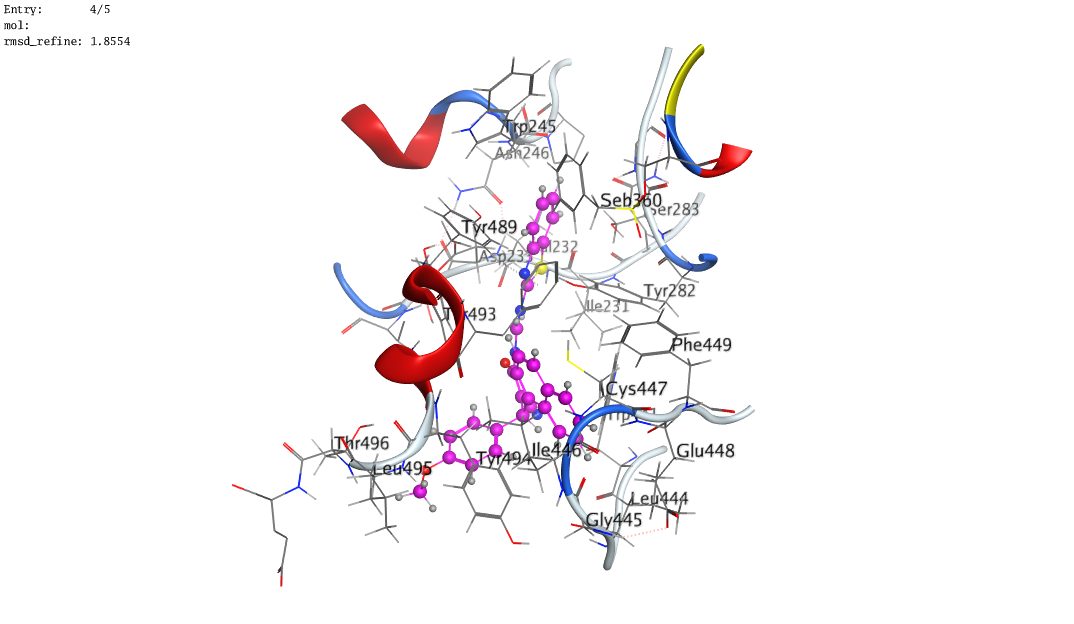 |
| --- | --- |
| **4**  **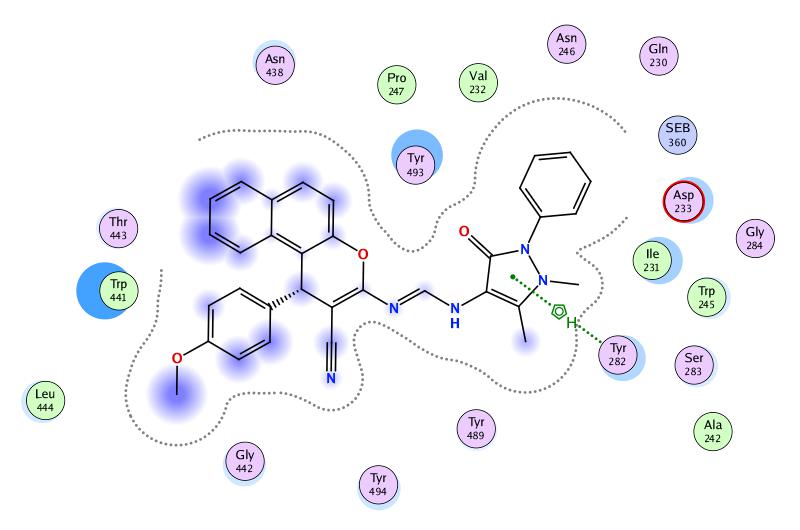** | 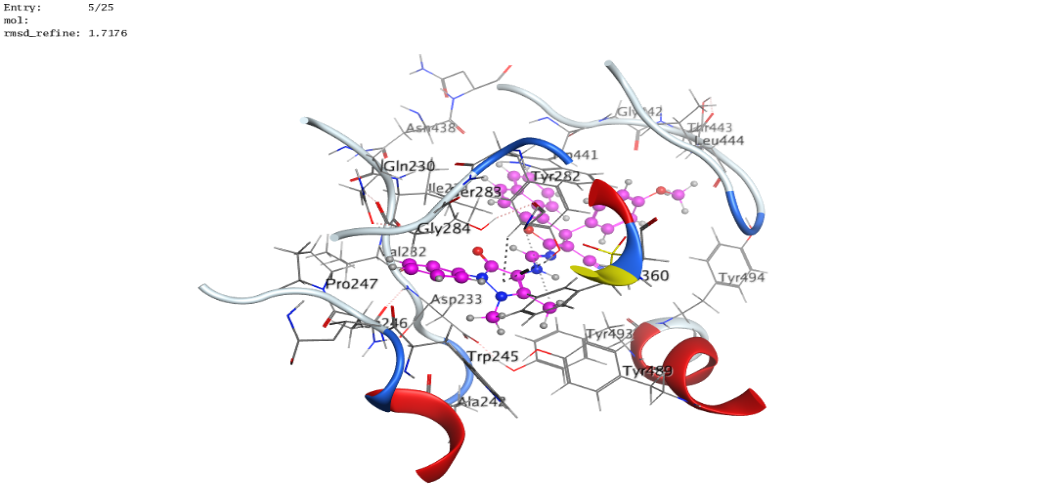 |
| **5**  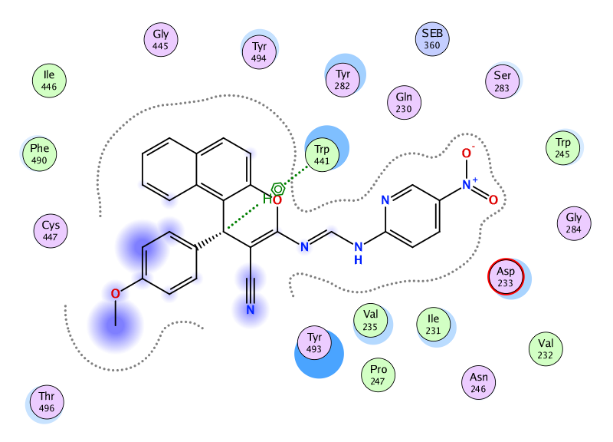 | 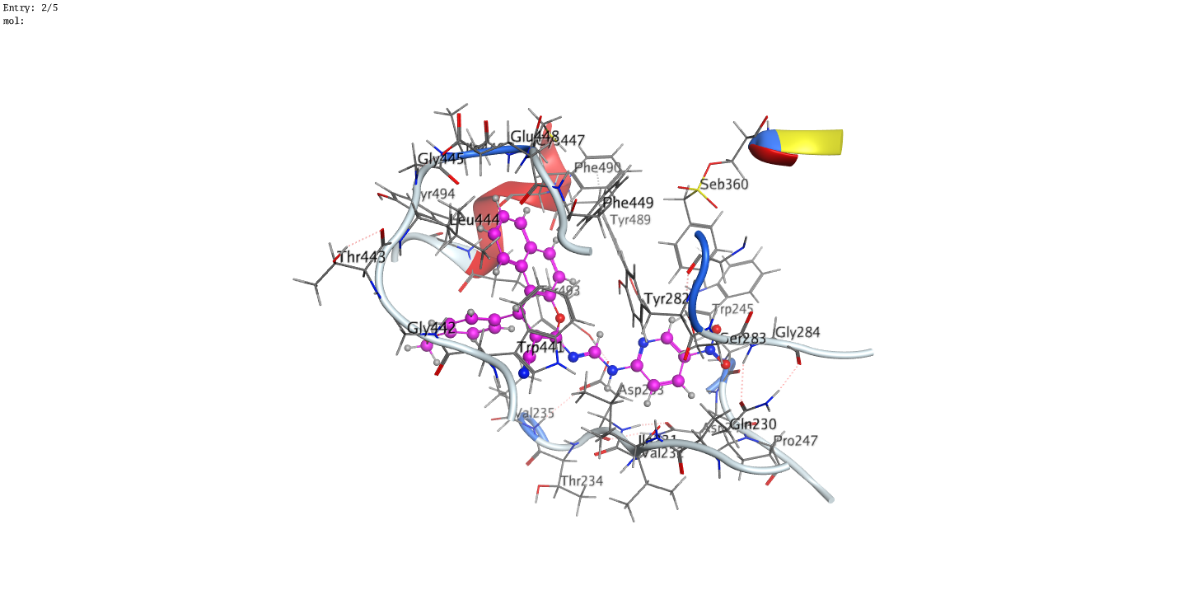 |
| **6**  **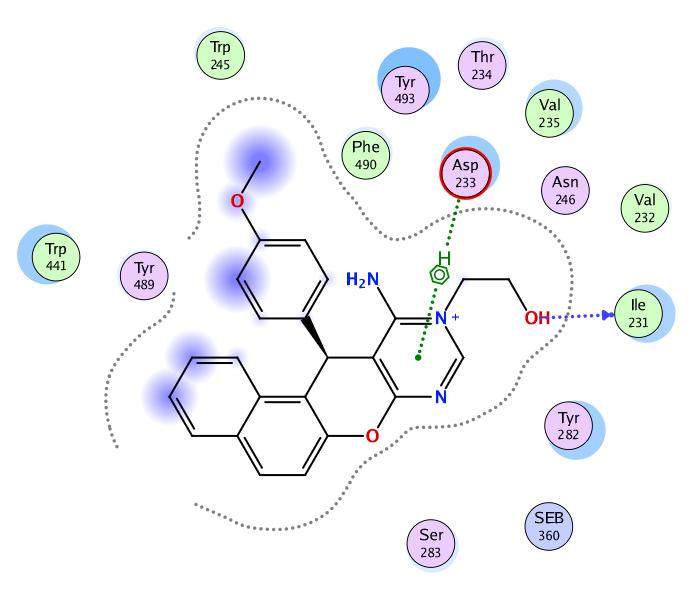** | 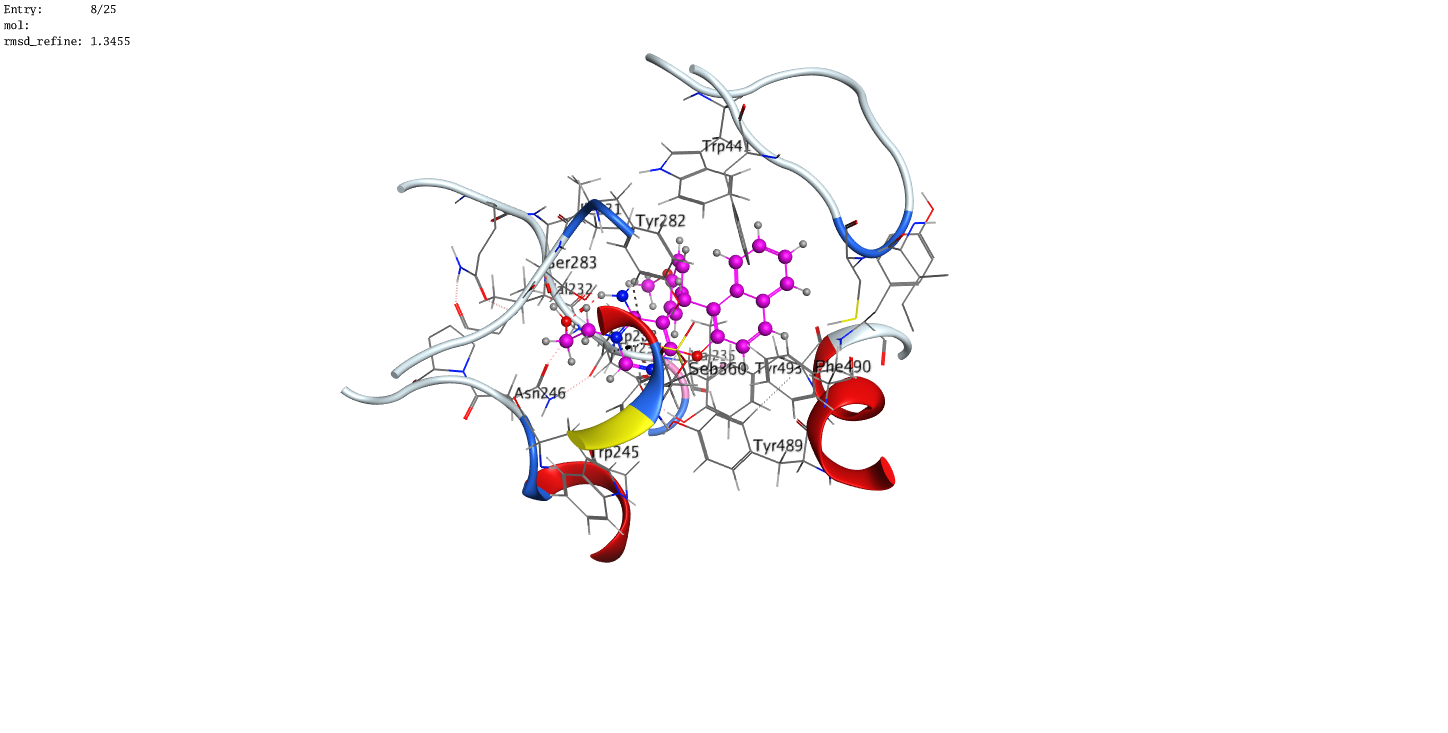 |
| **7**  **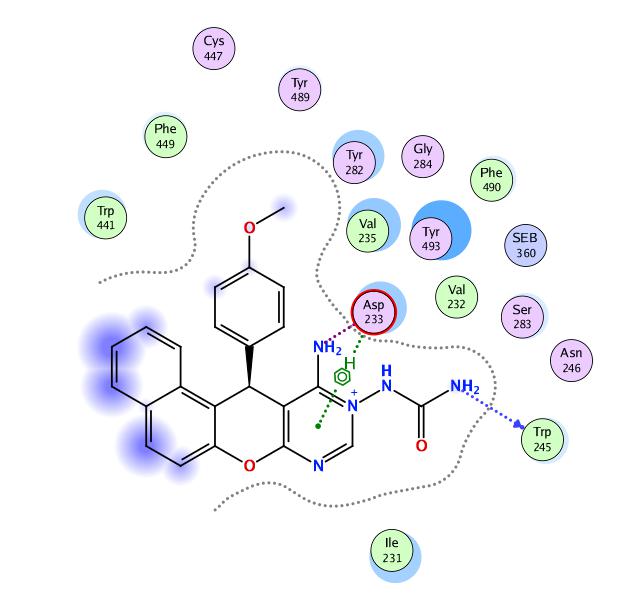** | 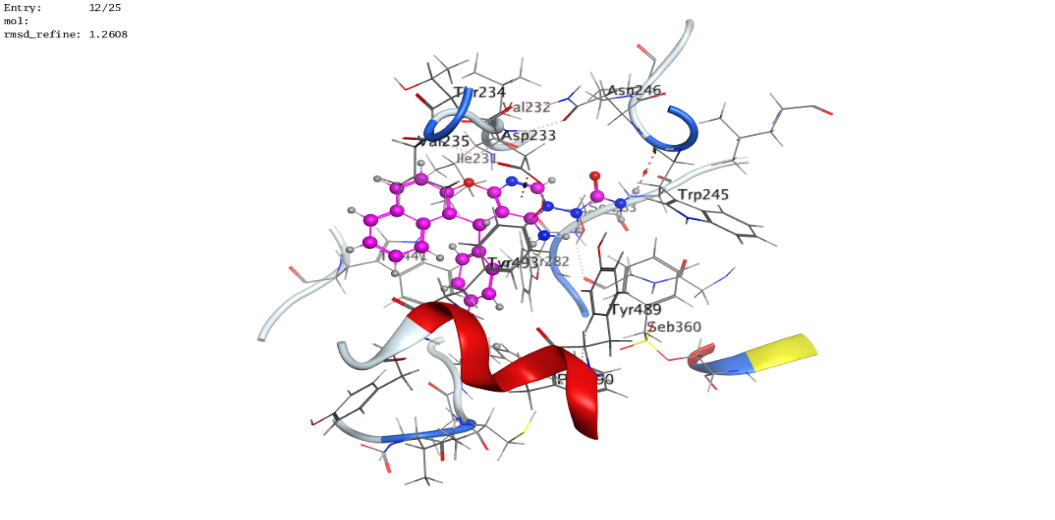 |
| **8**  **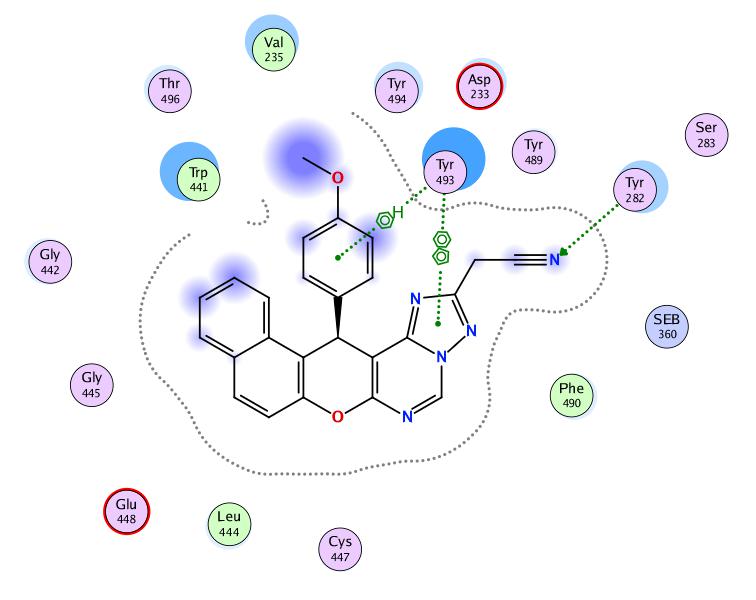** | 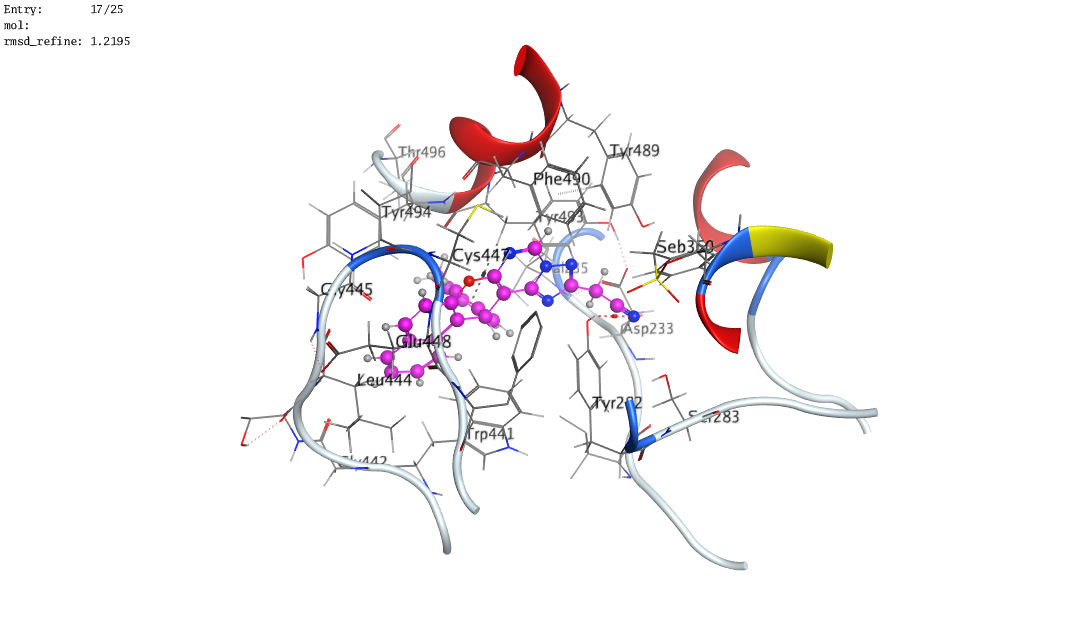 |
| **9**  **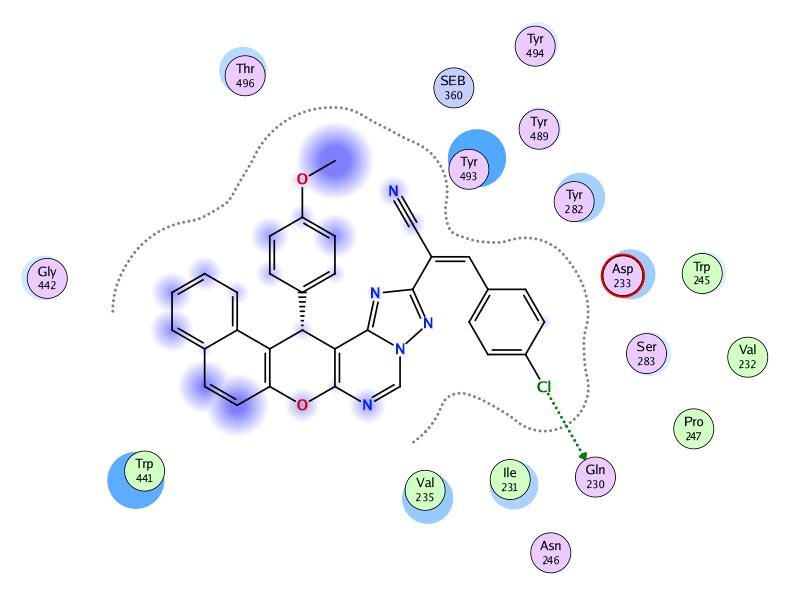** | 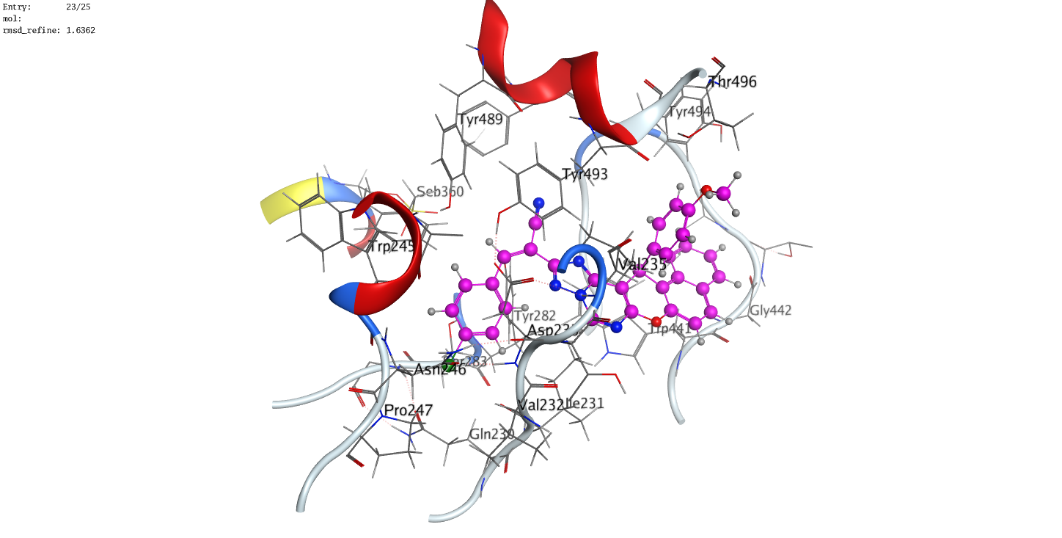 |
| **10**  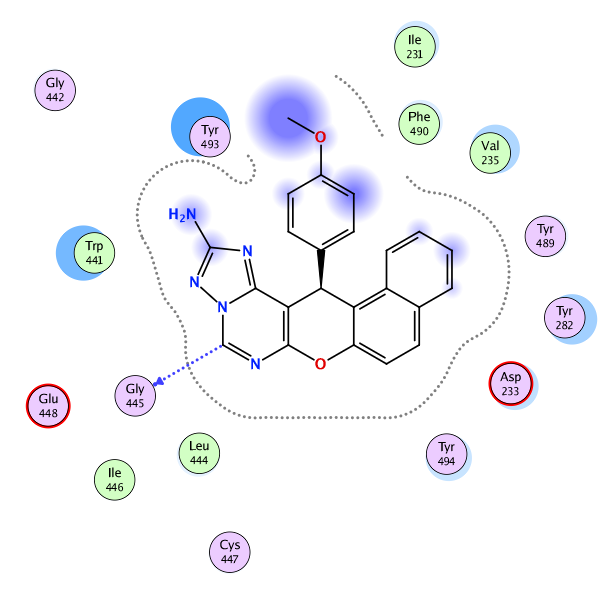 | 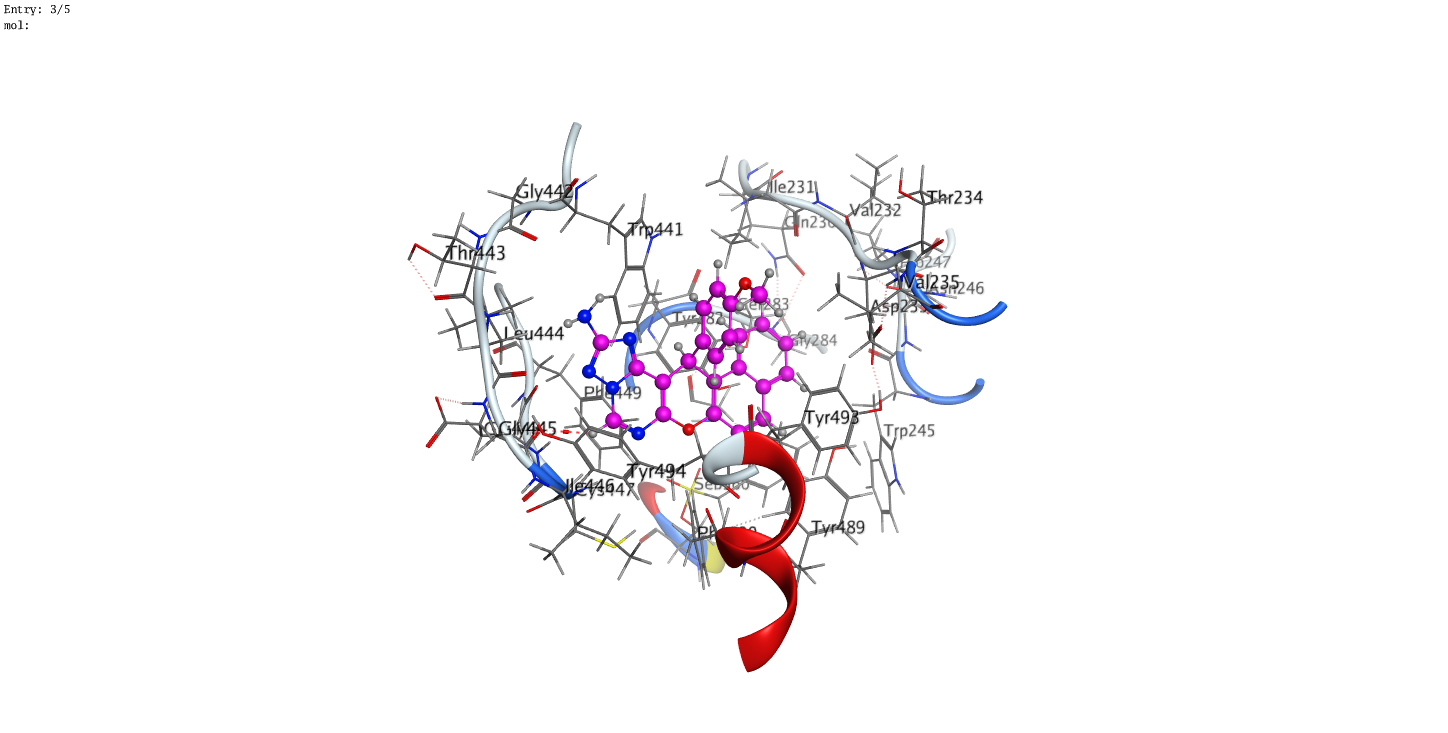 |
| **Temephos**  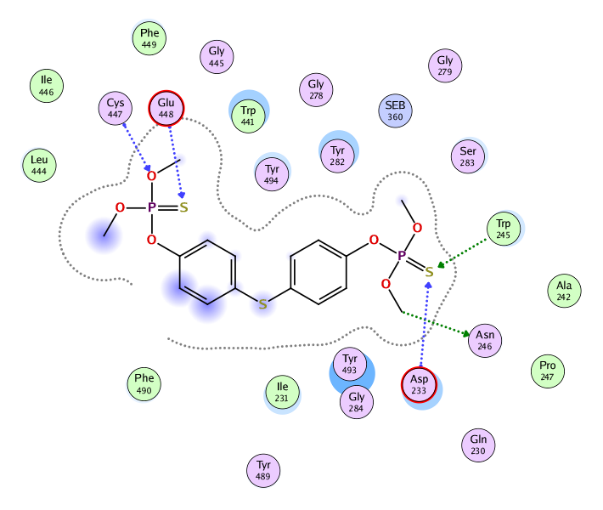 | 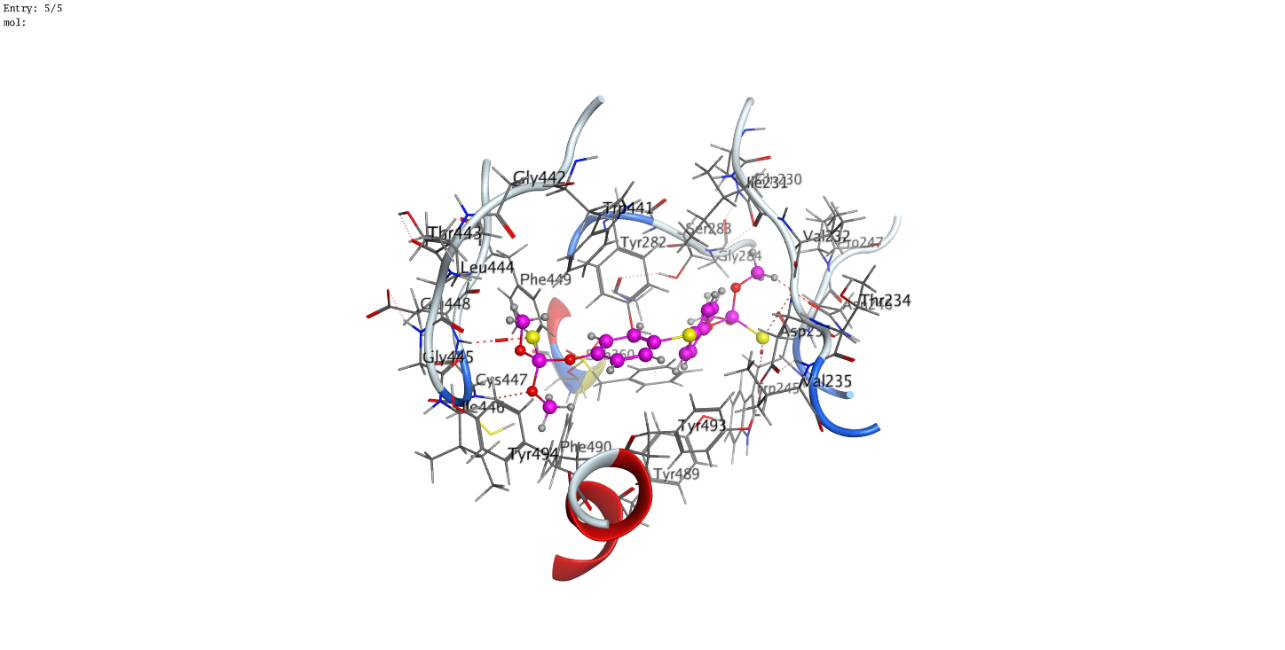 |
| **Ligand**  **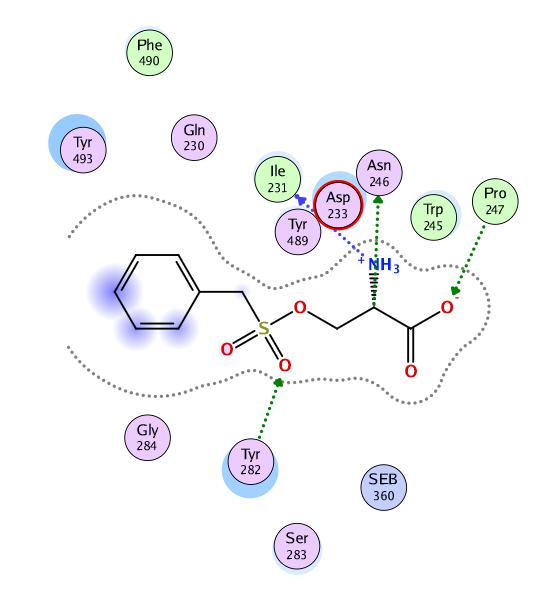** | 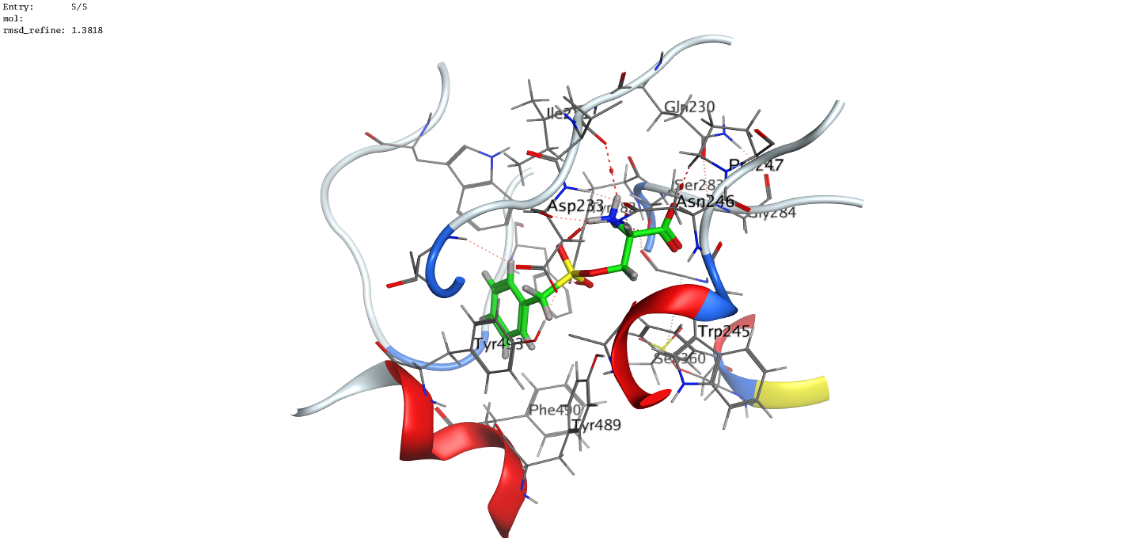 |

Fig. S48: 2D and 3D interaction visualization of tested compounds, temephos and ligand with the target site of *Anopheles gambiae* AChE (PDB code: 5YDJ).


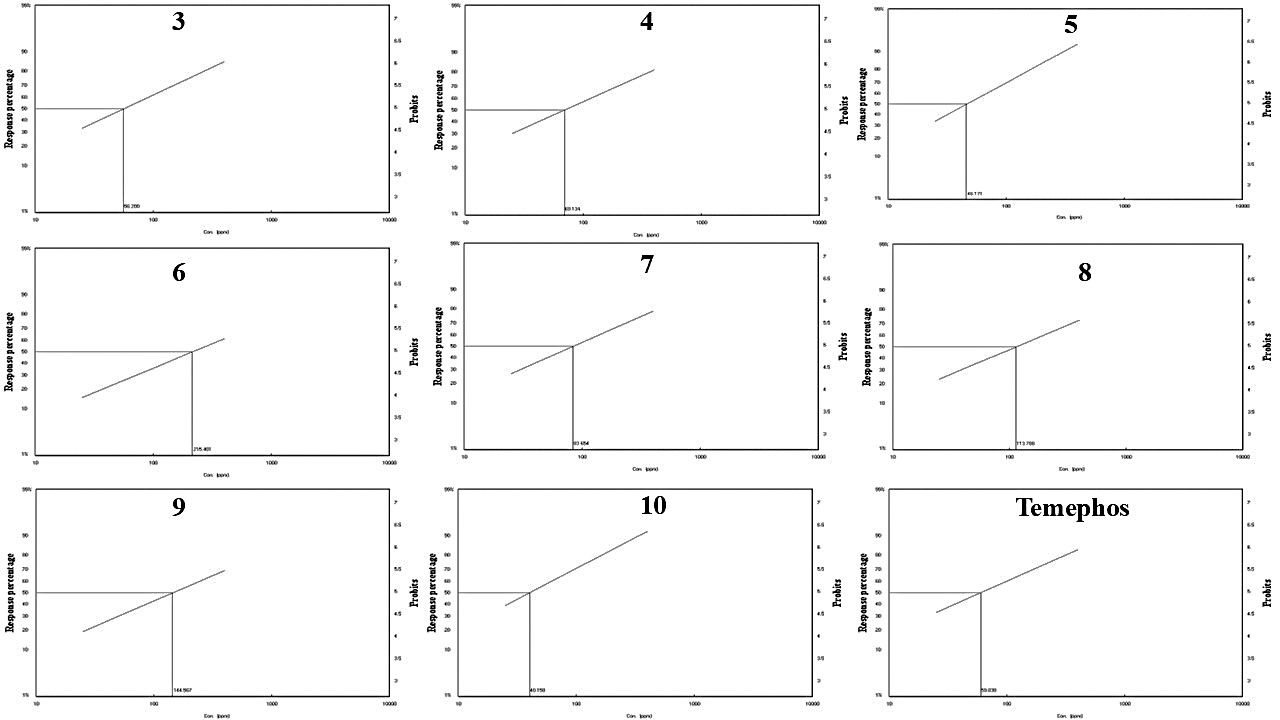


Fig. S49**:** Probit analysis graph showing regression lines and LC_50_ values of tested compounds against early third instar larvae of *Culex pipiens* at 48 h post-treatment estimated by the Finney probit method (Finney 1971).


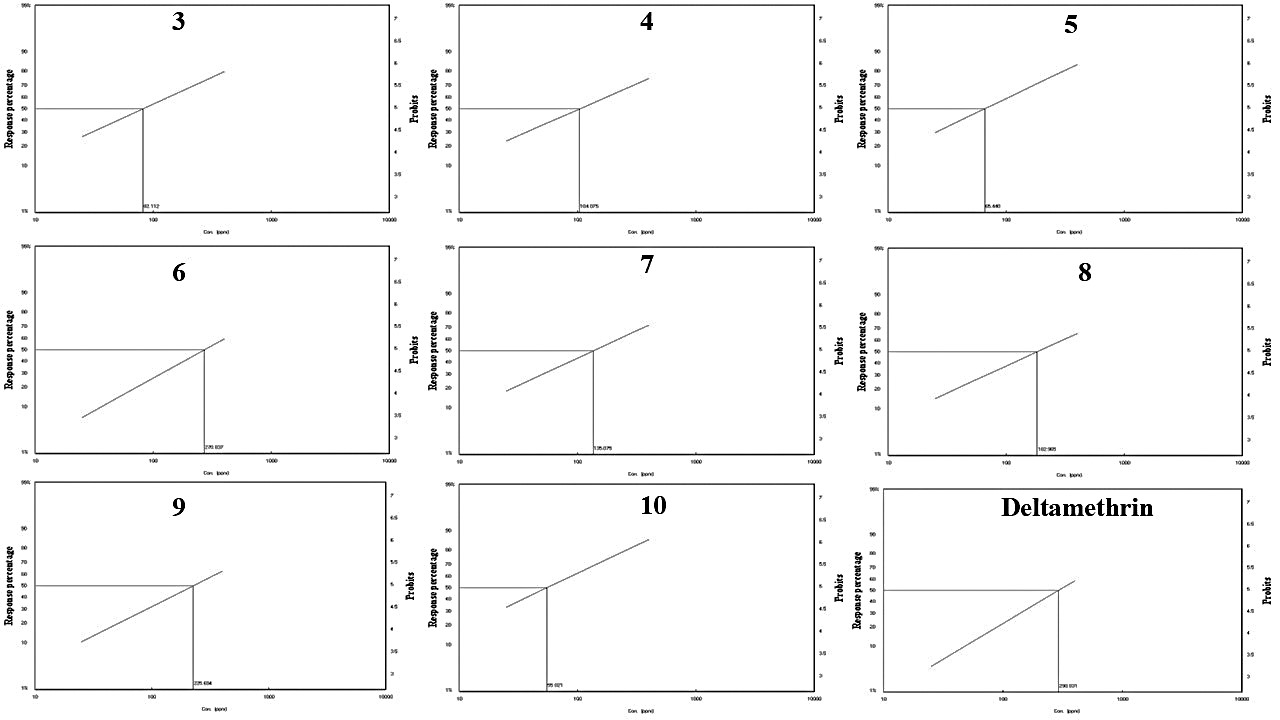


Fig. S50**:** Probit analysis graph showing regression lines and LC_50_ values of tested compounds against *Culex pipiens* adults at 24 h post-treatment estimated by the Finney probit method (Finney 1971).
